# Assessment of Intrathecal Free Light Chain Synthesis: Comparison of Different Quantitative Methods with the Detection of Oligoclonal Free Light Chains by Isoelectric Focusing and Affinity-Mediated Immunoblotting

**DOI:** 10.1371/journal.pone.0166556

**Published:** 2016-11-15

**Authors:** David Zeman, Pavlína Kušnierová, Zdeněk Švagera, František Všianský, Monika Byrtusová, Pavel Hradílek, Barbora Kurková, Olga Zapletalová, Vladimír Bartoš

**Affiliations:** 1 Institute of Laboratory Diagnostics, University Hospital Ostrava, 17. listopadu 1790, 708 52 Ostrava-Poruba, Czech Republic; 2 Clinic of Neurology, University Hospital Ostrava, 17. listopadu 1790, 708 52 Ostrava-Poruba, Czech Republic; 3 Institute for Healthcare Disciplines, St. Elisabeth University of Health and Social Work in Bratislava, Palackého 1, P.O. Box 104, 810 00 Bratislava, Slovak Republic; Istanbul University, TURKEY

## Abstract

**Objectives:**

We aimed to compare various methods for free light chain (fLC) quantitation in cerebrospinal fluid (CSF) and serum and to determine whether quantitative CSF measurements could reliably predict intrathecal fLC synthesis. In addition, we wished to determine the relationship between free kappa and free lambda light chain concentrations in CSF and serum in various disease groups.

**Methods:**

We analysed 166 paired CSF and serum samples by at least one of the following methods: turbidimetry (Freelite™, SPA_PLUS_), nephelometry (N Latex FLC™, BN ProSpec), and two different (commercially available and in-house developed) sandwich ELISAs. The results were compared with oligoclonal fLC detected by affinity-mediated immunoblotting after isoelectric focusing.

**Results:**

Although the correlations between quantitative methods were good, both proportional and systematic differences were discerned. However, no major differences were observed in the prediction of positive oligoclonal fLC test. Surprisingly, CSF free kappa/free lambda light chain ratios were lower than those in serum in about 75% of samples with negative oligoclonal fLC test. In about a half of patients with multiple sclerosis and clinically isolated syndrome, profoundly increased free kappa/free lambda light chain ratios were found in the CSF.

**Conclusions:**

Our results show that using appropriate method-specific cut-offs, different methods of CSF fLC quantitation can be used for the prediction of intrathecal fLC synthesis. The reason for unexpectedly low free kappa/free lambda light chain ratios in normal CSFs remains to be elucidated. Whereas CSF free kappa light chain concentration is increased in most patients with multiple sclerosis and clinically isolated syndrome, CSF free lambda light chain values show large interindividual variability in these patients and should be investigated further for possible immunopathological and prognostic significance.

## Introduction

Although the presence of free kappa light chains (fKLC) in the cerebrospinal fluid (CSF) of multiple sclerosis (MS) patients was already assumed in 1974 [1], only recently had they been widely advocated as a tool in the laboratory support of MS diagnosis or to estimate the probability of developing MS in patients after the first demyelinating event, the so-called clinically isolated syndrome (CIS) [2–10]. Already in the 1980s and 1990s, several qualitative [11–17] and quantitative [18–22] methods were introduced for the assessment of free kappa (fKLC) as well as lambda (fLLC) light chains in the CSF, but none of them have reached wide acceptance due to labouriousness and possibly also lack of standardisation. The development of turbidimetric/nephelometric free light chain (fLC) assays for serum analysis [23] and subsequent introduction of these tests into clinical practice for the diagnosis and monitoring of patients with plasma cell dyscrasias at the beginning of the 21st century [24, 25] opened a more convenient way to fLC quantitation not only in serum and urine, but also in CSF. Nevertheless, fLC concentrations in CSF are much lower than those in serum, which made the development of CSF assays quite challenging.

Already in 2004 Fischer et al. published a study on the use of Freelite™ for CSF fKLC measurement on Siemens BN II analyser [26]. One year later, Desplat-Jégo et al. [27] published the values for CSF fKLC as well as fLLC using the same reagents on the same analyser.The CSF fKLC concentration measured in the non-inflammatory subgroup was substantially higher, as compared to the previous study, and CSF fLLC levels were also surprisingly high in this subgroup. Until 2014, all of these adaptations of Freelite fLC assays had been off-label. Recently, The Binding Site Company has introduced CE marked kits designed for CSF fKLC as well as fLLC measurement. The same is hopefully expected in the near future for N Latex FLC kits by Siemens introduced for serum fLC measurement in 2011 [28].

However, several problems persist. First, there is still no internationally recognised primary standard. The source of most commercially available purified fLC is either serum or urine of patients with fLC paraproteinaemia or Bence Jones paraproteinuria, respectively. The Binding Site primary standards are light chains purified from intact immunoglobulins by reduction and acetylation, which has been criticised by Nakano et al., who proposed a purification procedure for polyclonal fLC from human urine as an alternative for standard preparation [29]. Normal serum values obtained using this standard were much higher (43.5 ± 12.0 mg/L for fKLC and 55.2 ± 17.9 mg/L for fLLC) than those obtained earlier by the same research group with the same ELISA method using purified human myeloma fLC standards (25.7 ± 10.5 mg/L and 4.34 ± 2.0 mg/L for fKLC and fLLC, respectively) [30]. Nevertheless, the values obtained by the same group using the same (monoclonal) standards but different ELISA method with two anti-fLC specific antibodies [31] gave even more different results (3.11 ± 1.18 mg/L and 2.30 ± 1.03 mg/L for fKLC and fLLC, respectively). These discrepancies might be related to the second problem of fLC assays: even minor cross-reactivities of the antibodies with bound light chains could result in significant overestimation of fLC in biological material where the concentration of immunoglobulins is approximately a thousand times larger than that of the fLC [32]. In addition, it has been shown that some antibodies employed in fLC assays detect better fLC dimers rather than monomers, while the degree of fLC dimerisation can vary under pathological conditions [33].

Third, all of the fLC tests have primarily been developed to help in the diagnosis and monitoring of plasma cell dyscrasias, while their performance in other settings has not yet been sufficiently validated.

Finally, fLC concentrations in normal CSF are often so low that they cannot be measured even by the modifications of the original assay. Even with latex particle enhancement, turbidimetry or nephelometry simply come to their limits on principal grounds at concentrations below 0.1 mg/L.

Reports on the diagnostic performance of CSF fKLC measurement in the context of MS diagnosis are extraordinarily consistent. Almost all studies showed performance similar to, or even marginally better than, the detection of oligoclonal IgG bands, irrespective of what the qualitative or quantitative methods were used and whether CSF fKLC concentration, CSF/Serum quotient, fKLC index (fKLC quotient/Albumin quotient) or any kind of non-linear relationship between fKLC quotient and albumin quotient was used for evaluation. The value of CSF fLLC measurement is less clear. While the earliest reports found intrathecal fLLC synthesis more frequently than that of fKLC [11, 12, 17], the reverse is observed in almost all later studies, with one notable exception [34]. Some groups were unable to develop a suitable fLLC assay [20, 35]. Other groups failed to report their fLLC results although they had apparently performed the CSF fLLC measurement [9] making one guess that the relative scarcity of fLLC data might be caused by some methodical problems and consequently a publication bias.

We therefore aimed at choosing the most suitable method for the routine detection of intrathecal fLC synthesis. After we had succesfully introduced oligoclonal fLC detection [36, 37], we looked for a quantitative method for comparison and possibly monitoring purposes. Our sandwich ELISA, which was developed during several years, is based on the same principle as the o-fLC test [38]. After being CE marked for CSF fLC analysis, we started to use the Freelite assay in our laboratory [39] and aimed to compare it with other commercially available or in-house developed tests. The present paper is intended to report the analysis of such a comparison and to point to some questions that arose during this research.

## Materials and Methods

### Patients and samples

Paired CSF and serum samples of 166 patients were included in the analysis. We categorised the patients into diagnosis groups as follows: 1. multiple sclerosis (MS; n = 17), 2. clinically isolated syndrome (CIS; n = 30), 3. other inflammatory neurological diseases (OIND; n = 20), 4. non-inflammatory neurological diseases (NIND; n = 57), 5. no evidence of organic nervous system disease (symptomatic controls according to the definition of Teunissen *et al*. [40]; n = 18), and 9. unknown or uncertain diagnoses (n = 24). Three CIS patients had asymptomatic gadolinium-enhancing lesions on brain magnetic resonance imaging (MRI) defining definite MS in the latest version of McDonald’s diagnostic criteria [41] at the time of lumbar puncture. Nevertheless, the patients were still categorised within the CIS group for the purpose of this study because they experienced the first attack of the disease shortly before the time of lumbar puncture. Other eight CIS patients progressed into definite MS during the period of sample recruitment (May 2014 –August 2015) or data analysis (September–October 2015). The diagnoses in the OIND group comprised meningoencephalitis (n = 3), encephalitis (n = 1), acute disseminated encephalomyelitis (n = 1), myelitis (n = 2), neuroborreliosis (n = 2), neurosyphilis (n = 1), neurolupus (n = 1), neuromyelitis optica spectrum disease (n = 1), plexitis (n = 1) and immune-mediated polyneuropathies (n = 7).

Patients with MS together with those CIS patients that fulfilled the recent definite MS criteria at the time of lumbar puncture or had progressed into definite MS before the diagnosis data were collected and analysed formed the MS group (n = 28) to be compared with the non-MS group consisting of patients of the groups 3, 4 and 5 (n = 95). For the comparison between inflammatory neurological diseases (IND) and non-inflammatory neurological diseases (NIND), we compared the MS group described above plus patients of the group 3 (n = 48) against patients in the groups 4 and 5 (n = 75). CIS patients that did not progress into definite MS during the study period as well as patients with an unknown or uncertain diagnosis were excluded from these analyses.

CSF was drawn by lumbar puncture and venous blood by venipuncture under standard conditions. Within one week after CSF withdrawal, immediately after all routine tests including oligoclonal IgG detection had been performed, paired CSF and serum samples were frozen to -30°C and thawed only once just before the fLC quantitative analysis. CSF storage at +2 to +8°C for up to 7 days is allowed in the package insert of the Freelite SPA_PLUS_ kits (method A, see below). Additionally, we have tested 11 CSF samples frozen either immediately or after 7 days at +2°C to +8°C and found only marginal decrease of fKLC concentrations and no decrease of fLLC concentrations (detailed data are available from Dryad, doi: 10.5061/dryad.8tk26). For oligoclonal fLC detection, an aliquot was kept at +2 to +8°C for up to 20 days. We have previously reported that oligoclonal fLC patterns remain stable during this period [37].

All the patients signed the informed consent for anonymised use of the surplus of the fluids for research purposes. The study was approved by the Ethics Committee of the University Hospital Ostrava (Ref. No. 319/2014).

### Methods

#### Free light chain quantitative assays

(A) Turbidimetric assay on SPA_PLUS_ with Freelite Kappa SPA_PLUS_ kit and Freelite Lambda SPA_PLUS_ kit (Cat. No. LK016.L.S and LK018.L.S, respectively, The Binding Site Ltd., Birmingham, UK). These tests use polyclonal sheep antibodies coated onto polystyrene latex. Calibrators are serum-based. The assay was performed according to the manufacturer’s instructions. CSF is analysed neat and if the concentration measured exceeds that of the highest standard, the analysis is repeated after manual dilution 1/10. The assay uses an extrapolated calibration curve with triplicate measurement of blank and standard 1 (lot-specific, about 0.35 mg/L for fKLC and 0.45 mg/L for fLLC), enabling measurement down to 0.1 mg/L. Values lower than 0.1 mg/L were analysed as 0.09 mg/L in this study. The highest calibrator has concentration about 16 mg/L (lot-specific) for both fKLC and fLLC.

(B) Nephelometric assay on BN ProSpec with N Latex FLC kit (Siemens Healthcare Diagnostics Products GmbH, Marburg, Germany, catalogue numbers OPJA03 –OPJF03). The assay uses a mixture of mouse monoclonal antibodies covalently coupled to polystyrene particles. Standards contain purified polyclonal fLC in PBS with 1% human serum albumin [28]. Based on our preliminary experiments, the lowest calibration point was deleted for CSF fKLC analysis on our request by the Siemens technician in order to avoid too many repetitive dilutions. Then the calibration curve spanned the range between 0.0616 and 1.97 mg/L for CSF fKLC, 0.0308 and 0.985 mg/L for Serum fKLC and 0.104 and 3.32 mg/L for both CSF and Serum fLLC. CSF is analysed neat and as soon as the signal exceeds that of the highest standard, automatic dilutions 1/5, 1/20 and 1/100 are performed by the instrument until the measured signal falls within that of the standards. Serum is diluted 1/100 for fKLC and 1/20 for fLLC analysis.

(C) Human Immunoglobulin Free Light Chains Kappa and Lambda ELISA kit (BioVendor-Laboratorni medicina a.s., Brno, Czech Republic, Cat. No. RD194088100R). The wells are pre-coated with monoclonal anti-human immunoglobulin fKLC or fLLC antibodies. The standards are human serum based. CSFs were diluted 1/2 (or more, if appropriate) and sera 1/200. The test was performed according to manufacturer´s instructions. Both standards and samples were analysed in duplicate. The manufacturer provides no information concerning the source of antibodies and it is not clear whether the second antibody is anti-free light chain specific or whether it detects free as well as bound light chains. Six calibrators prepared by subsequent two-fold dilutions span the concentration range 10–320 μg/L for fKLC and 17.5–560 μg/L for fLLC.

(D) In-house ELISA. Microtiter plates (F96MaxiSorp F96, Nunc A/S, Roskilde, Denmark, Cat. No. 442404) were coated either with 0.75 mg/L anti-human free kappa light chain antibody (DAKO, Glostrup, Denmark, Cat. No. A0100) or 0.30 mg/L anti-human free lambda light chain antibody (DAKO, Cat. No. A0101) diluted in carbonate-bicarbonate buffer, pH 9.5 ± 0.1. The plates were covered with paraffin foil and incubated for 18–48 hours in 4°C. Next, plates were washed twice with phosphate-buffered saline (PBS; pH 7.3±0.1)-0.05% Tween 20 (PBST) and blocked with 1% (w/v) bovine serum albumin (BSA, fraction V receptor grade; Serva Electrophoresis GmbH, Heidelberg, Germany, Cat. No. 11924) in PBST for 75 minutes. CSF and serum were diluted with 0.2% (w/v) BSA in PBST. Usual dilution was 1/10 for CSF and 1/1000 for serum. Samples with high or low fLC content were diluted as appropriate in order for the signal to fall within the calibration curve. For calibration, monoclonal free light chains (Bethyl Laboratories, Montgomery, Texas, USA, Cat. No. P080-126 and P080-127) were used. Seven two-fold dilutions (concentration range between 1.25 and 80.00 μg/L for fKLC and 2.5 and 160.0 μg/L for fLLC) were prepared. The plate was washed 5 times with PBST. Standards and samples were applied in duplicate and the plate was incubated for 2 hours in room temperature. Next, the plate was washed 5 times with PBST, while the biotinylated anti-fLC antibody prepared as described earlier [36] diluted 1/10,000 (0.4 mg/L) in 0.2% BSA. After 90-minute incubation, decantation and washing, horseradish peroxidase streptavidin (ELISA grade, Vector Laboratories, Burlingame, CA, USA, Cat. No. SA-5014) diluted 1/750 was applied and the plate was incubated for 40 minutes and washed as described above. Finally, TMB one-step substrate system (DAKO, Cat. No. S159985) was applied and the plate was incubated until suitable colour intensity developed (18–20 minutes). After stopping the reaction with 50 μl of 1M H_2_SO_4_, the optical density was read by DSX ELISA automatic analyser (Dynex, Buštěhrad, Czech Republic). The 4-parameter calibration curve was constructed using the analyser software.

(E) In-house ELISA as described in (D) but using appropriately diluted (1/200) Freelite standards 1–6. The 4-parameter calibration curve was constructed using the in-house developed programme. Calibration range was about 1.8 and 80 μg/L for fKLC and 2.25 and 80 μg/L for fLLC with slight lot-to-lot variations.

Our sandwich ELISA was developed during several years [38] and uses a principle that is similar to the method of qualitative detection of oligoclonal fLC bands by means of affinity-mediated immunoblotting, described by Sindic and Laterre [16] and later modified by us [36, 37]. Both intra- and interassay coefficients of variations (CVs) were determined for Freelite control samples. In addition, interassay CVs were determined in several pooled CSF and serum samples. Recovery was determined by spiking CSF and serum samples with 10 vol.% of either Bethyl monoclonal light chains or Freelite serum-based standard. Details concerning analytical characteristics of the in-house methods are available from Dryad, doi: 10.5061/dryad.8tk26

#### Detection of oligoclonal fLC (reference method)

Isoelectric focusing (IEF) in 1.2% agarose gels containing 12% sorbitol (interelectrode distance 8.5 cm, limits 200 V/cm, 100 mA, 10 W) was carried out on Multiphor II apparatus (GE Healthcare UK Ltd., Buckinghamshire, England) at 10°C for 1,200 Vh. 7.5 μl of the respective monoclonal fLC protein (Bethyl, Cat. No. P080-126 for fKLC and P080-127 for fLLC) diluted to 2.0 and 0.2 mg/L were run as positive controls, while intravenous immunoglobulin preparation (Flebogamma, Instituto Grifols S.A., Barcelona, Spain, or Kiovig, Baxter AG, Wien, Austria) diluted to 250 or 500 mg/L IgG was run as a negative control. CSF samples were used neat and serum samples were diluted 1/100. All dilutions were performed with 0.75% saline. Paired CSF and serum samples were applied side by side using applicator strips (Serva, Cat. No. 42899). After IEF, fLC were blotted onto nitrocellulose membranes (Amersham™ Hybond ECL, Cat. No. RPN303D, or Amersham™ Protran™ Premium, Cat. No. 10600003, GE Healthcare, pore size 0.45 μm) precoated with anti-fLC antibodies (DAKO, Cat. No. A0100 for fKLC and A0101 for fLLC) and blocked with 3% BSA in tris-buffered saline (75 mM TBS, pH 7.5 ± 0.1) for 60 minutes. Next, membranes were washed briefly in phosphate-buffered saline, followed by the fixation of proteins on the membrane with 0.25% glutardialdehyde for 15 minutes at 4°C. Then, the membranes were briefly rinsed in deionised H_2_O, washed 3 times in TBS, re-blocked with 0.3% BSA in TBS for 15–20 minutes, washed once with TBS and incubated with biotin-labelled anti-fLC antibody (diluted to 5 mg/L) for 105 ± 5 minutes. After washing once in TBS, 3 times in TBST and 2 times in TBS, incubation followed with Alkaline phosphatase streptavidin (Vector, USA, Cat. No. SA-5100) diluted 1/750 for 50 minutes. Next, the membranes were washed twice in TBS, 3 times in TBST and 2 times in TBS. Finally, colour reaction was developed using Alkaline Phosphatase Substrate IV Kit–BCIP/NBT (Vector, USA, Cat. No. SK-5400) for 30 minutes. Membranes were then washed in TBS for 5 minutes and in deionised water for 1–2 minutes. After being held for 1 minute in a vertical position to let the water drop out, the membranes were dried overnight between 2 sheets of filter paper covered with a glass plate.

### Statistical analysis

Statistical analysis was performed using MedCalc statistical software version 14.12.0 (MedCalc software bvba, Ostend, Belgium; http://www.medcalc.org; 2014). Kolmogorov-Smirnov test was used for the assessment of normality whenever appropriate. Differences between the methods were assessed by means of Spearman´s correlation coefficient, log-log scatter diagrams, Passing and Bablok regression and Bland-Altman plots. ROC curves were analysed and compared, if appropriate, for the prediction of a positive o-fLC test as well as the MS or IND diagnosis. Correlations between categorical variables (o-IgG and o-fLC) were investigated by the Chi-square test.

## Results

### Oligoclonal IgG and oligoclonal fLC results

The results of these tests in patients within individual diagnosis groups, MS/non-MS and IND/NIND groups are provided in S1 Table. Only 1 out of 92 patients negative for o-fKLC was positive for o-IgG and o-fLLC. Out of 74 patients positive for o-fKLC, 18 were o-IgG negative and 22 were o-fLLC negative. Out of 53 patients positive for o-fLLC, 8 were o-IgG negative. The correlations between the results of o-IgG, o-fKLC and o-fLLC tests were significant (chi-square test, all P values <0.0001). Representative examples of o-fLC detection using our IEF/AIB method are shown in S1 Fig (the original blots from which this figure was combined are available from Dryad, doi:10.5061/dryad.8tk26.

### Quantitative method comparison

**Fig 1:**
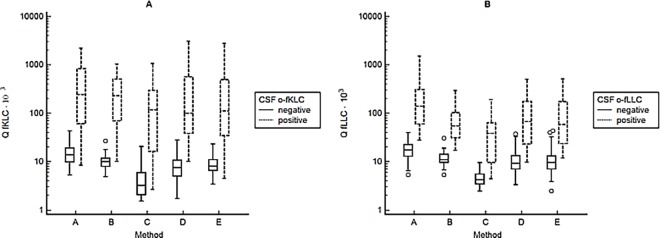
CSF/Serum quotients of fKLC and fLLC determined by methods (A)-(E).

The comparison of CSF/Serum quotients determined by various methods is presented in Fig 1. For in-depth comparison of CSF and serum concentrations as well as CSF/Serum quotients see Tables A-C in S1 File, Figures A-F in S2 File and Figures A-F in S3 File. Both systematic and proportional differences occurred. The largest differences were observed between the results of the commercially available ELISA method in the CSF that were much lower than those of all other tests. Nevertheless, the obtained concentrations were comparable with those published previously [9]. Serum fLLC were measured much higher by nephelometry using N Latex FLC kit than by turbidimetry using Freelite assay, although there was no significant difference between the two methods when measuring fLLC in CSF.

To get some insight into the possible reasons of differences among the methods used, we analysed standards used in the individual assays by the in-house ELISA method. Optical densities of the standards diluted to similar fLC concentrations differed substantially, especially in the case of fKLC standards. For more details see the S2 Fig.

### Correlation between fLC quotient and albumin quotient

In the group of patients with negative o-fKLC test (<2 CSF-restricted fKLC bands, n = 87), a clear correlation could be seen between fKLC quotient and albumin quotient (r = 0.6028, 95% CI 0.4491–0.7218, P<0.0001), while the absence of such a correlation was obvious in the CSF o-fKLC positive group (n = 70, r = -0.1322, 95% CI -0.3561–0.1060, P = 0.2752). Also, the presence of the correlation between fLLC quotient and albumin quotient in the o-fLLC negative group (n = 109, r = 0.5171, 95% CI 0.3645–0.6427, P<0.0001) and the absence of this correlation in the o-fLLC positive group (n = 49, r = -0.04943, 95% CI = -0.3261–0.2350, P = 0.7359) was obvious (S3 Fig).

### ROC curves for the prediction of a positive o-fLC test

ROC curves for the prediction of a positive CSF-restricted o-fLC test showed AUCs >0.9 except for fKLC index using ELISA BioVendor assay and fLLC index using all the three ELISAs employed with AUCs 0.87 in all these four cases. There was no significant difference in the AUCs between CSF concentrations, CSF/Serum quotients or indices. When the cut-off for CSF-restricted o-fKLC bands was raised to ≥6 bands as in our previous studies [37, 39], all AUCs for CSF fKLC, fKLC quotient as well as fKLC index were >0.95. For the Freelite method on SPA_PLUS_, calculated cut-off values were >0.78 mg/L for CSF fKLC, >43.19 ∙ 10^3^ for fKLC quotient and >6.07 for fKLC index, i. e. similar to those found in our preliminary study [39]. However, we feel that while searching for optimal cut-offs may be important for diagnostic purposes, the canonical definition of ≥2 CSF-restricted oligoclonal bands is rather appropriate for a methodical study. The optimal cut-off values calculated by the ROC curve analysis differed substantially among the methods (Table 1).

**Table 1:** Cut-offs for predicting positive o-fLC test (≥2 CSF-restricted bands) suggested by receiver operating characteristic curve analysis.

Free kappa light chains	*n (o-fKLC+/ o-fKLC-)*	*CSF fKLC (mg/l)*	*fKLC quotient (∙10*^*3*^*)*	*fKLC index*
Freelite™ on SPA_PLUS_	70/89 (70/87 for fKLC quotient and index)	>0.54 (82.9; 97.8)	>30.61 (84.3; 98.9)	>3.25 (90.0; 82.8)
N Latex FLC™ on BN ProSpec	20/29	>0.417 (85.0; 100)	>17.60 (90.0; 96.6)	>3.21 (80.0; 100)
ELISA (BioVendor)	22/16	>0.025 (90.9; 87.5)	>9.09 (81.8; 93.8)	>1.20 (77.3;87.5)
ELISA (in-house, monoclonal standards)	49/84 (46/82 for fKLC quotient and index)	>0.216 (87.8; 90.5)	>19.18 (89.1; 95.1)	>2.75 (84.8; 96.3)
ELISA (in-house, Freelite™ standards)	56/85	>0.340 (85.7; 94.1)	>17.48 (89.3; 96.5)	>2.17 (89.3; 89.4)
**Free lambda light chains**	***n (o-fLLC+/ o- fLLC-)***	***CSF fLLC (mg/l)***	***fLLC quotient (∙10***^***3***^***)***	***fLLC index***
Freelite™ on SPA_PLUS_	49/110 (49/109 for fLLC quotient and index)	>0.30 (98.0; 91.8)	>36.31 (91.8; 99.1)	>6.68 (79.6; 97.3)
N Latex FLC™ on BN ProSpec	15/33	>0.368 (93.3; 87.9)	>16.92 (100; 90.9)	>3.82 (86.7; 100)
ELISA (BioVendor)	17/21	>0.039 (94.1; 90.5)	>7.38 (82.4; 95.2)	>0.92 (82.4;80)
ELISA (in-house, monoclonal standards)	40/102	>0.358 (87.5; 91.2)	>22.05 (77.5; 96.1)	>3.03 (75.0; 91.2)
ELISA (in-house, Freelite™ standards)	40/102	>0.272 (92.5; 85.3)	>21.11 (80.0; 92.2)	>2.88 (77.5; 90.2)

n, number of positive/negative cases; o-, oligoclonal; fKLC, free kappa light chains; fLLC, free lambda light chains. Sensitivity and specificity obtained by receiver operating characteristic analysis are given in parentheses. All areas under the curve were above 0.9 except for fKLC index using BioVendor ELISA and fLLC index using BioVendor ELISA as well as in-house ELISAs (all these areas under the curve were 0.87). At pairwise comparison of other assays against the Freelite™ assay, in-house ELISAs for CSF fLLC and fLLC quotient performed somewhat worse (P = 0.0167–0.0434); all other differences between the areas under the curve were not significant (P > 0.05).

Further, the ROC curves for the Freelite™ method on SPA_PLUS_ were compared pairwise with the ROC curves for all the other methods. The AUCs were significantly larger for the Freelite™ method on SPA_PLUS_ than for in-house ELISA using either monoclonal or polyclonal fLC standard only in the case of fLLC CSF concentration and quotient.

### ROC curves for the prediction of MS or inflammatory neurological disease diagnoses

**Fig 2:**
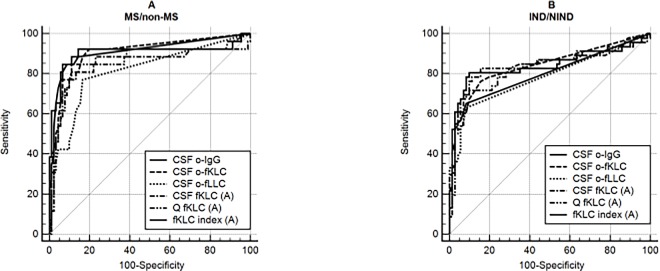
ROC curves in the context of MS and IND diagnoses. **a. MS versus non-MS b. Inflammatory neurological diseases versus non-inflammatory neurological diseases and symptomatic controls** a. 26 MS and 91 non-MS patients were compared. AUCs for o-IgG, o-fKLC, o-fLLC, CSF fKLC, Q fKLC and fKLC index were 0.911, 0.907, 0.813, 0.850, 0.857 and 0.903, respectively. Significant differences were found between o-IgG and o-fLLC (P = 0.0035), o-fKLC and o-fLLC (P = 0.0075), fKLC index and Q fKLC (P = 0.0031), o-fKLC and CSF fKLC (P = 0.0201), o-fKLC and Q fKLC (P = 0.0219), fKLC index and CSF fKLC (P = 0.0347). Differences between o-IgG and Q fKLC (P = 0.0477), o-IgG and CSF fKLC (P = 0.0700), and fKLC index and o-fLLC (P = 0.0474) were of borderline significance. Other differences were not significant. b. 46 inflammatory neurological diseases (IND) and 71 non-inflammatory neurological diseases and symptomatic controls (NIND) patients were compared. AUCs for o- IgG, o-fKLC, o-fLLC, CSF fKLC, Q fKLC and fKLC index were 0.798, 0.840, 0.783, 0.838, 0.825 and 0.840, respectively. Although the AUCs for o-fKLC and fKLC index are slightly larger than for o-IgG, the only significant difference was observed between o-fKLC and o-fLLC (P = 0.0299). Quantitative measurements by the method (A) (Freelite™on SPA_PLUS_) were considered for these comparisons.

Concerning the diagnostic performance of quantitative fLC data in the context of MS or inflammatory neurological disease (IND) diagnoses, fKLC AUCs were significantly larger than those of fLLC. Especially in the context of MS diagnosis, the use of fLC indices was shown to be superior to CSF concentrations or even CSF/Serum quotients. No significant difference between the diagnostic performance of the best quantitative fKLC measures, o-fKLC and o-IgG was revealed (Fig 2). Using the classical criterion of ≥2 CSF-restricted oligoclonal bands, sensitivities and specificities for MS were 85.7 and 88.4% for o-IgG, 92.9 and 72.6% for o-fKLC, and 64.3 and 84.2% for o-fLLC. For the diagnosis of IND, sensitivities and specificities were 62.5 and 93.3% for o-IgG, 77.1 and 85.3% for o-fKLC and 62.5 and 93.3% for o-fLLC. The optimal cut-off values of the quantitative tests calculated from the ROC curves again differed among methods (S2 and S3 Tables).

### fKLC/fLLC ratios in CSF and sera

**Fig 3:**
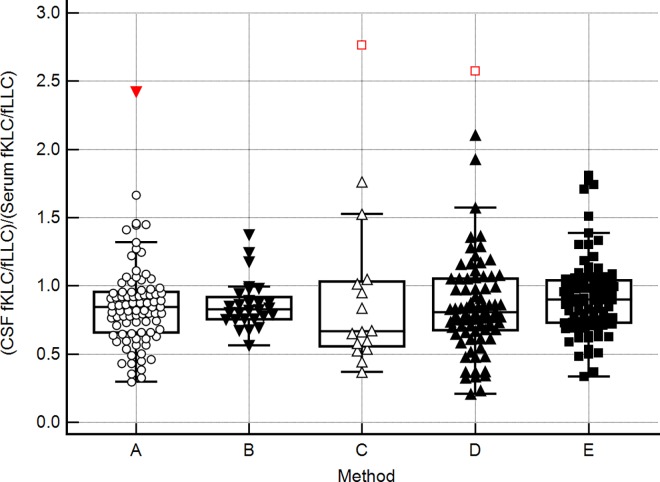
**Ratio (CSF fKLC/fLLC)/(Serum fKLC/fLLC) in patients without CSF-restricted o-fLC bands, using the methods (A)–(E).** CSF, cerebrospinal fluid; fLC, free light chains; fKLC, free kappa light chains; fLLC, free lambda light chains. (A), Freelite™ assay on the SPA_PLUS_ analyser; (B) N Latex FLC™ assay on BN ProSpec analyser; (C) commercially available ELISA (BioVendor); (D), in-house ELISA using monoclonal standards (Bethyl Laboratories); (E), in-house ELISA using Freelite™ standards.

**Fig 4:**
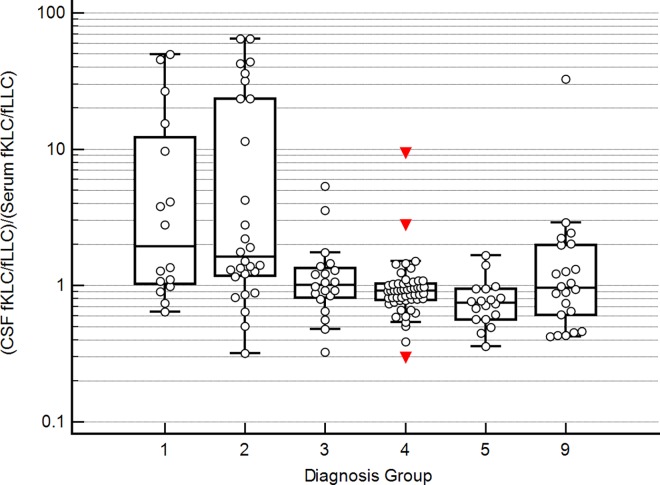
Ratio (CSF fKLC/fLLC)/(Serum fKLC/fLLC) in individual diagnosis groups, using the Freelite™ method on SPA_PLUS_. 1, multiple sclerosis; 2, clinically isolated syndrome; 3, other inflammatory neurological diseases; 4, non-inflammatory neurological diseases; 5, symptomatic controls; 9, uncertain/unknown diagnosis. It can be seen that about a half of multiple sclerosis and clinically isolated syndrome patients have an increased CSF/Serum quotient of fKLC/fLLC ratios compared to symptomatic controls.

In the absence of intrathecal fLC synthesis, CSF fKLC/fLLC ratios were slightly lower than the corresponding serum ratios. This result was consistent in all 5 used methods. Thus CSF/Serum quotient of these ratios [(CSF fKLC/fLLC)/(Serum fKLC/fLLC)] was slightly below 1 and the value of 1.0 corresponded approximately to the 75th percentile (Fig 3). About half of MS and CIS patients have a profoundly increased CSF fKLC/fLLC ratio normalised to serum fKLC/fLLC ratio as discussed above (Fig 4).

## Discussion

### Can CSF/Serum fLC quotients be regarded as method-independent values?

According to Reiber [42], CSF/Serum immunoglobulin quotients are method-independent values, provided that the concentrations in both fluids are measured within the same run on the same calibration curve. Nevertheless, despite good overall correlation of the data, we observed considerable differences between fLC quotient values, implying that different and method-specific cut-offs have to be used for the assessment of intrathecal synthesis. We conclude that given current analytical possibilities, quotient method-independence does not apply for fLC.

### Relationship between Q fLC and Q Albumin

We confirmed our earlier observations [39] that for both fKLC and fLLC there is a clear correlation between fLC quotient and albumin quotient values in cases with a negative o-fLC test. Conversely, no such a correlation was observed in cases with CSF-restricted o-fLC bands. This demonstrates that compared to the intrathecal fraction, the relative amount of serum-derived fLC fraction in the CSF becomes negligible, which is in line with the recent study of Presslauer et al. [8] where fKLC intrathecal fraction was close to 100% in most MS patients. This probably accounts for the fact that simple CSF fLC concentration has similar diagnostic sensitivity and specificity as quotients and indices, although we still recommend the use of the latter because false positive results of CSF fLC may occur in cases of very high serum fLC concentrations (e.g. in patients with paraproteinaemia or systemic inflammation). The relationship between fLC quotient and albumin quotient appears to be non-linear and the intrathecal synthesis could be calculated using either inverted Reiber’s hyperbole, as we proposed recently [39], or the power function proposed by Presslauer et al. [7, 8]. We demonstrated that almost all values lie above the line of equality, as expected for the CSF/Serum quotient of a smaller molecule plotted on the ordinate and that of a larger molecule plotted on the abscissa [43]. The shape of the curve displaying the non-linear relationship is also consistent with the theoretical expectations. However, more patients with negative o-fLC results, especially those with severe blood-CSF barrier disturbance, should be examined so that reliable parameters of these equations could be calculated.

### fKLC/fLLC ratios in CSF and sera

Besides CSF/Serum quotient, also fKLC/fLLC ratio can be calculated from the data. Since this ratio is used in serum as an aid in diagnosing and monitoring patients with monoclonal gammopathies, it might be interesting to detect a skewed distribution of fLC in CSF.

Our results are in accordance with older studies that showed substantially higher CSF fLLC than fKLC concentrations in the control groups [19, 21] but theoretically unexpected. fKLC are assumed to circulate predominantly as monomers, while fLLC as dimers. Hence, fKLC monomers with a lower molecular weight (22.5 kDa) should diffuse into the CSF more easily than fLLC dimers (45 kDa) and, consequently, the values above 1.0 should be obtained. The discrepancy between expected and real data suggests that either there is a general methodological problem in fLC analyses or that there might be a more complex monomer/dimer/oligomer fLC balance than it is currently percieved. Another explanation includes the presence of other physiological factors hindering fKLC or facilitating fLLC diffusion from blood into the CSF. Monomer/dimer patterns of fLC were studied in serum as well as in the CSF [33, 44, 45] and although these tests seem to be too complicated to be introduced into clinical routine, in further research studies they should complement quantitative determinations of fLC in order to solve this interesting question.

Since the intrathecal fLLC synthesis is usually accompanied by the fKLC synthesis, it may seem logical to drop fLLC studies and perform only fKLC tests. However, from the immunological point of view, the analysis of both fLC might be useful as widely different fLLC values were seen in MS patients by our group [39] as well as by the others [3,4]. It is plausible to suggest that the fKLC/fLLC ratio may provide important additional information on the intrathecal humoral immune response and might even be of prognostic significance. The skewed CSF fKLC/fLLC ratio may be the result of receptor editing/receptor revision [46, 47] that has already been described as occurring within the CNS of MS patients [48].

### Choosing the method: Practical considerations

Each method as well as each analyser has its own limits and advantages. Finally, we chose the Freelite turbidimetric assay on SPA_PLUS_ analyser, which is economic and user-friendly; manual dilution of the CSF sample is rarely required for fKLC and almost never for fLLC. Nevertheless, ELISA based assays might be better applicable in some laboratories worldwide where access to automated instruments is limited. In addition, diluted CSF is used in our in-house ELISA assay, which leads to a much lower amount of sample required for the analysis. Although the sample volume in the automatised assays is low (SPA_PLUS_ requires 15 μl for fKLC and 6.5 μl for fLLC analysis, i.e. 21.5 μl for both measurements, while 50 μl–i.e. 100 μl for both measurements–were required for the analysis on BN ProSpec), we have to add the dead volume that is slightly above 100 μl in both cases. Nephelometric method on BN ProSpec (with either Freelite or N Latex FLC) might be best suited for scientific purposes. Besides having marginally better sensitivity for fKLC (about 0.05 mg/L versus 0.1 mg/L on the SPA_PLUS_ instrument), in consequent dilutions it strives to achieve a signal in the sample that is close to the middle of the calibration curve where the measurement can be expected to be most precise. On the other hand, this results in a high consumption of reagents for samples with high CSF fLC concentrations, which is a major drawback of this method.

### Limits of the study

We are aware of several weaknesses of our study. First, it was impossible to perform all tests on every sample, mainly due to insufficient amounts of CSFs and/or sera needed for the assays. Testing by the N Latex FLC method on BN ProSpec had to be interrupted prematurely because of quality control failure regarding a new lot of reagents that was unfortunately only discovered after thawing a proportion of samples. Simultaneously performing in-house ELISA methods with monoclonal and polyclonal (Freelite™) standards, we found out there were rare cases when one sample showed a signal higher than that of the highest standard in one method, but within the calibration range in the other method. However, the analysis could not be repeated in higher dilution either due to insufficient amount of sample or for logistic reasons.

Second, although theoretically sound, the assumption that the detection of CSF-restricted oligoclonal immunoglobulin or fLC bands represents a gold standard to prove intrathecal synthesis, against which quantitative measures should be compared, was only sufficiently validated for IgG. The negative result of oligoclonal IgG detection is often used as a reference method for detecting the absence of intrathecal humoral immune reaction, but it was shown that o-fLC could sometimes be detected in the absence of o-IgG, especially in other inflammatory CNS diseases rather than MS [16, 37]. Therefore, we tried to validate CSF fLC quantitation against o-fLC detection as the reference method. However, unlike for IgG, the amounts of fLC in paired CSF and sera are not matched in the o-fLC assays published so far (including that of ours). Instead, neat CSF and arbitrary diluted serum (1/100) are used. This approach seems more appropriate than the dilution of serum and CSF to the same IgG concentration used in several older studies [11, 12, 16] because fLC have a smaller molecular weight and therefore a considerably larger serum fraction passes into the CSF. In patients with severe blood-CSF-barrier disturbance, however, systemic o-fLC response might still be falsely detected as intrathecal if serum fLC concentrations are not elevated. In our experience, however, systemic o-fLC response is detected much less frequently than systemic o-IgG response.

## Conclusions

In spite of considerable proportional as well as systematic differences between various quantitative fLC assays compared in this study, no major differences were observed regarding the prediction of a positive o-fLC test or the diagnostic performance in the context of inflammatory nervous system disease diagnosis. The performance of o-fKLC as well as quantitative fKLC tests is comparable to that of o-IgG, but not superior. In the context of MS diagnosis, o-IgG seems to be more specific than fKLC tests. We therefore conclude that it is too early to recommend abandoning the o-IgG test as suggested in some recent reports [3, 8] until more data have been gathered from various laboratories and different fKLC assay methods have been used. The diagnostic performance of qualitative as well as quantitative fLLC tests is inferior to that of o-IgG and fKLC tests, but enormous variability in the CSF fLLC concentration observed among individual MS patients warrants further studies including prospective evaluation of the possible significance of intrathecal fLLC synthesis in MS. Besides, the fKLC/fLLC ratio in normal CSFs seems to be lower than theoretically expected, based on the postulated predominance of circulating fKLC monomers and fLLC dimers and on the Reiber’s blood-CSF barrier diffusion theory [49], which remains to be elucidated.

## Supporting Information

S1 Fig Oligoclonal fKLC (left) and fLLC (right) detected by isoelectric focusing followed by affinity-mediated immunoblotting. ST2 and ST0.2, fKLC and fLLC standards (2.0 and 0.2 mg/L, i.e. 15 and 1.5 ng) as positive controls; F, IgG preparation (Flebogamma, 500 mg/L) as a negative control; C1+S1, C2 + S2: CIS (case 1 subsequently converted into definite MS). Both CSF samples are positive for fKLC, while only the second sample is positive for fLLC. CSF fKLC concentrations were 5.09 and 1.74 mg/L, while CSF fLLC concentrations 0.10 and 1.06 mg/L, respectively.C3 + S3: myelitis of unknown aetiology (diagnosis group 3); both fKLC and fLLC OCBs can be seen in the CSF. CSF concentration of fKLC was 2.02 mg/L and that of fLLC 1.37 mg/L.At control 7 months later, no fLC bands were seen and the CSF fLC concentrations fell to normal (CSF fKLC 0.12 mg/L, CSF fLLC 0.18 mg/L). Control sample was not included in the analysis.C4 + S4: cryptogenic oculomotor mononeuropathy (diagnosis group 4); normal CSF, negative result of both o-fKLC and o-fLLC (CSF fKLC 0.14 mg/L, CSF fLLC 0.24 mg/l) Anode is at the top.
(GIF)

S2 Fig **Standards of methods (A)–(D) analysed by the in-house ELISA method.**Calibrators of methods (A)–(D) together with Freelite® calibrators for serum assays (Catalogue Numbers LK016.S and LK018.S, respectively, marked A (S) in the figure legend) were diluted to similar fLC concentrations and analyzed by in-house ELISA (D). Please note that method (E) used the same calibrator as method (A). Dilution curves were compared. Optical densities (OD) were very similar for both Freelite® calibrators. Otherwise, however, there were considerable differences among the calibrators, especially for fKLC.
(PDF)

S3 Fig Relationship between fLC quotient and albumin quotient. A clear correlation can be seen between fLC quotients and albumin quotients in the group of o-fLC negative samples, whereas no such correlation is observed when there is intrathecal fLC synthesis.
(TIFF)

S1 File Comparison of CSF and serum fLC concentrations measured by methods (A)—(E) and CSF/Serum fLC quotients by means of Passing and Bablok regression and Spearman´s correlation coefficient. **Table A. Comparison of CSF fLC concentrations measured by methods (A)—(E) by means of Passing and Bablok regression and Spearman´s correlation coefficient**. (A), Freelite™ assay on the SPA_PLUS_ analyser; (B) N Latex FLC™ assay on BN ProSpec analyser; (C) commercially available ELISA (BioVendor); (D), in-house ELISA using monoclonal standards (Bethyl Laboratories); (E), in-house ELISA using Freelite™ standards; fLC, free light chains; fKLC, free kappa light chains; fLLC, free lambda light chains; CI, confidence interval **Table B. Comparison of serum fLC concentrations measured by methods (A)—(E) by means of Passing and Bablok regression and Spearman´s correlation coefficient**. (A), Freelite™ assay on the SPA_PLUS_ analyser; (B) N Latex FLC™ assay on BN ProSpec analyser; (C) commercially available ELISA (BioVendor); (D), in-house ELISA using monoclonal standards (Bethyl Laboratories); (E), in-house ELISA using Freelite™ standards; fLC, free light chains; fKLC, free kappa light chains; fLLC, free lambda light chains; CI, confidence interval **Table C. Comparison of CSF/Serum fLC quotients by means of Passing and Bablok regression and Spearman´s correlation coefficient**.(A), Freelite™ assay on the SPA_PLUS_ analyser; (B) N Latex FLC™ assay on BN ProSpec analyser; (C) commercially available ELISA (BioVendor); (D), in-house ELISA using monoclonal standards (Bethyl Laboratories); (E), in-house ELISA using Freelite™ standards; fLC, free light chains; fKLC, free kappa light chains; fLLC, free lambda light chains; CI, confidence interval
(ZIP)

S2 File CSF and serum fLC concentrations and CSF/Serum fLC quotients. **Method comparison (scatter diagrams). A. CSF fKLC (mg/L) B. Serum fKLC (mg/L) C. CSF fLLC (mg/L) D. Serum fLLC (mg/L) E. Q fKLC (∙ 10^3^) F. Q fLLC (∙ 10^3^)** CSF, cerebrospinal fluid; fLC, free light chains; fKLC, free kappa light chains; fLLC, free lambda light chains; Q, CSF/Serum quotient. (A), Freelite™ assay on the SPA_PLUS_ analyser; (B) N Latex FLC™ assay on BN ProSpec analyser; (C) commercially available ELISA (BioVendor); (D), in-house ELISA using monoclonal standards (Bethyl Laboratories); (E), in-house ELISA using Freelite™ standards
(ZIP)

S3 File Bland-Altman plots. **A. CSF fKLC (mg/L) B. Serum fKLC (mg/L) C. CSF fLLC (mg/L) D. Serum fLLC (mg/L) E. Q fKLC (∙ 10^3^) F. Q fLLC (∙ 10^3^)** CSF, cerebrospinal fluid; fLC, free light chains; fKLC, free kappa light chains; fLLC, free lambda light chains; Q, CSF/Serum quotient. (A), Freelite™ assay on the SPA_PLUS_ analyser; (B) N Latex FLC™ assay on BN ProSpec analyser; (C) commercially available ELISA (BioVendor); (D), in-house ELISA using monoclonal standards (Bethyl Laboratories); (E), in-house ELISA using Freelite™ standards.
(ZIP)

S1 Table Results of o-IgG, o-fKLC and o-fLLC tests (≥2 CSF-restricted bands). **a. according to the diagnosis group b. according to MS and IND status** MS, multiple sclerosis; CIS, clinically isolated syndrome; OIND, other (than MS) inflammatory neurological diseases; NIND, non-inflammatory neurological diseases; IND, inflammatory neurological diseases.
(RTF)

S2 Table Cut-offs in the context of multiple sclerosis diagnosis. **a. Free kappa light chains b. Free lambda light chains** MS, multiple sclerosis; non-MS, other diagnoses than multiple sclerosis or clinically isolated syndrome It should be noted that the number of samples examined by the N Latex FLC™ and BioVendor ELISA is too small to permit definitive conclusions regarding the performance of these tests in the context of MS diagnosis.* The second best cut-off value in case the calculated cut-off resulted in ≤50% sensitivity or specificity that was considered as unacceptable.
(RTF)

S3 Table Cut-offs in the context of inflammatory neurological disease diagnosis. **a. Free kappa light chains b. Free lambda light chains** IND, inflammatory neurological diseases; NIND, non-inflammatory neurological diseases and controls * The second best cut-off value in case the calculated cut-off resulted in ≤50% sensitivity or specificity that was considered as unacceptable. ** The second best cut-off value in case the calculated cut-off resulted in ≤50% specificity that was considered as unacceptable.
(RTF)
