# Supplementary figures and images for: Assessment of Intrathecal Free Light Chain Synthesis: Comparison of Different Quantitative Methods with the Detection of Oligoclonal Free Light Chains by Isoelectric Focusing and Affinity-Mediated Immunoblotting
Source: PLoS One. 2016 Nov 15;11(11):e0166556. doi: 10.1371/journal.pone.0166556 (PMC5112955; doi:10.1371/journal.pone.0166556)

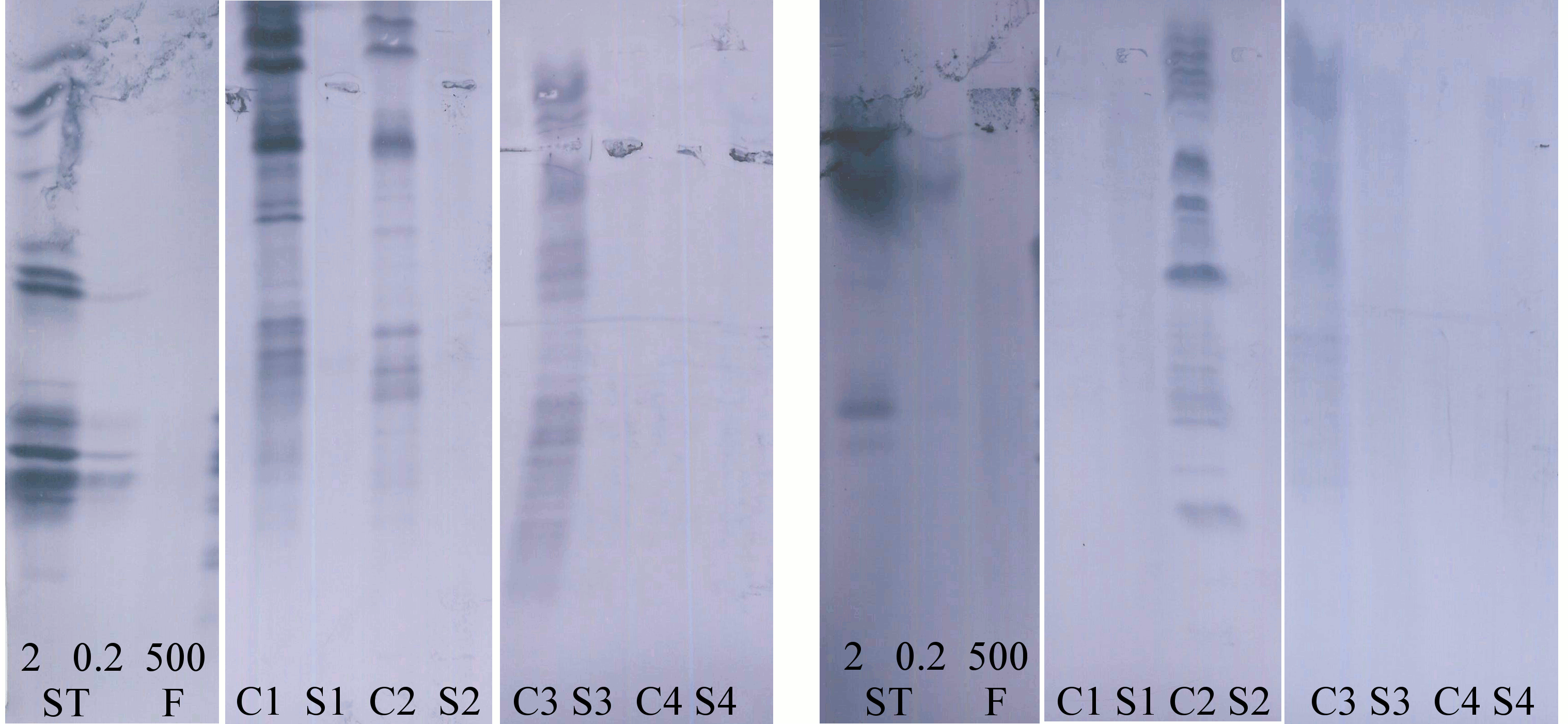

Supplement: S1 Fig — ST2 and ST0.2, fKLC and fLLC standards (2.0 and 0.2 mg/L, i.e. 15 and 1.5 ng) as positive controls; F, IgG preparation (Flebogamma, 500 mg/L) as a negative control; C1+S1, C2 + S2: CIS (case 1 subsequently converted into definite MS). Both CSF samples are positive for fKLC, while only the second sample is positive for fLLC. CSF fKLC concentrations were 5.09 and 1.74 mg/L, while CSF fLLC concentrations 0.10 and 1.06 mg/L, respectively.C3 + S3: myelitis of unknown aetiology (diagnosis group 3); both fKLC and fLLC OCBs can be seen in the CSF. CSF concentration of fKLC was 2.02 mg/L and that of fLLC 1.37 mg/L.At control 7 months later, no fLC bands were seen and the CSF fLC concentrations fell to normal (CSF fKLC 0.12 mg/L, CSF fLLC 0.18 mg/L). Control sample was not included in the analysis.C4 + S4: cryptogenic oculomotor mononeuropathy (diagnosis group 4); normal CSF, negative result of both o-fKLC and o-fLLC (CSF fKLC 0.14 mg/L, CSF fLLC 0.24 mg/l) Anode is at the top. (GIF) [file pone.0166556.s001.gif]

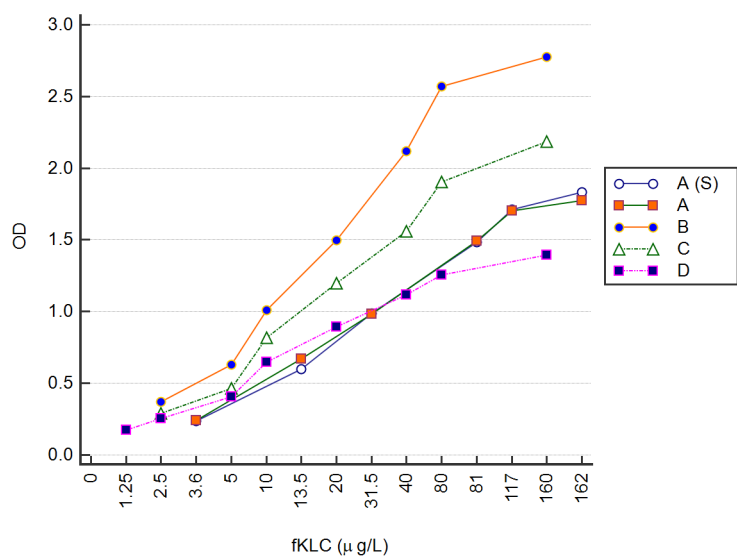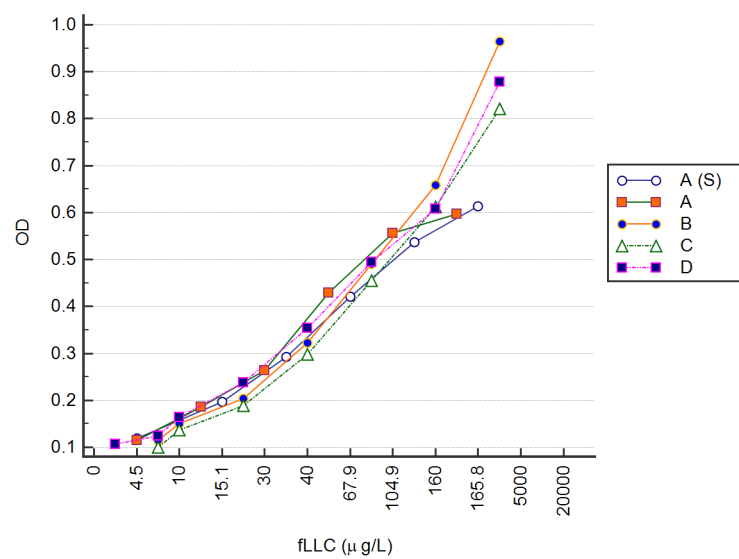

Supplement: S2 Fig — Standards of methods (A)–(D) analysed by the in-house ELISA method.Calibrators of methods (A)–(D) together with Freelite® calibrators for serum assays (Catalogue Numbers LK016.S and LK018.S, respectively, marked A (S) in the figure legend) were diluted to similar fLC concentrations and analyzed by in-house ELISA (D). Please note that method (E) used the same calibrator as method (A). Dilution curves were compared. Optical densities (OD) were very similar for both Freelite® calibrators. Otherwise, however, there were considerable differences among the calibrators, especially for fKLC. (PDF) [file pone.0166556.s002.pdf]

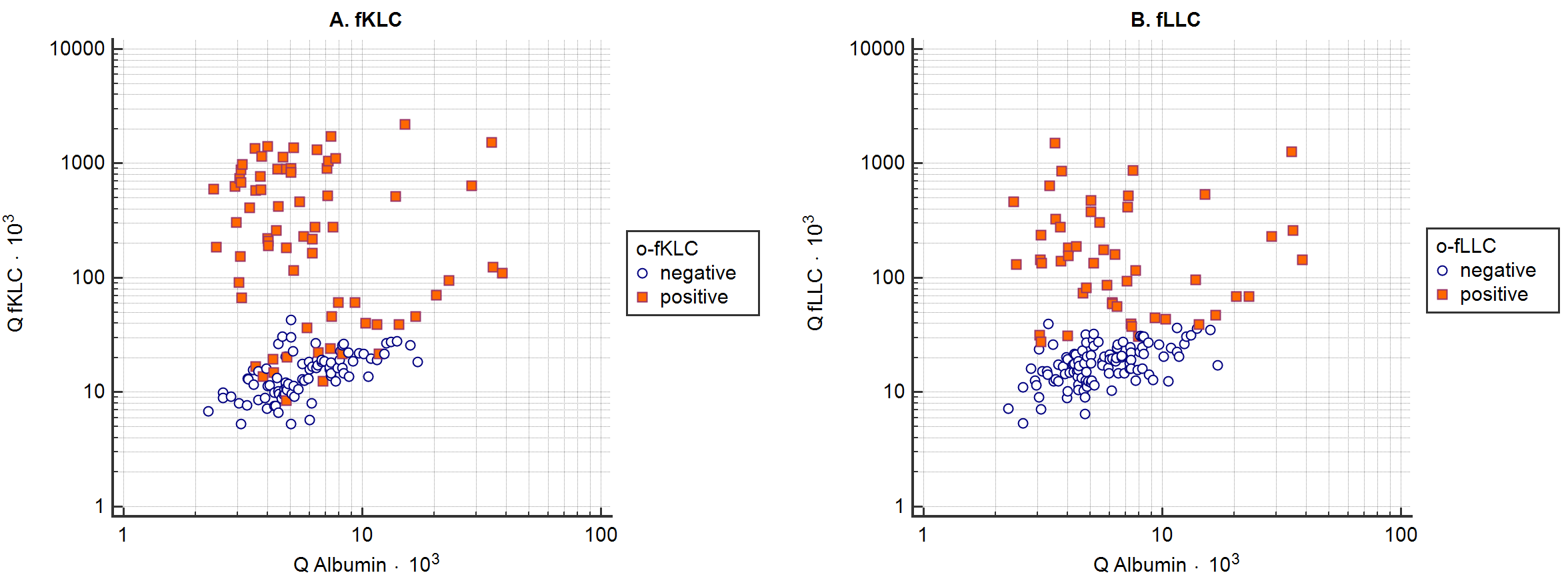

Supplement: S3 Fig — A clear correlation can be seen between fLC quotients and albumin quotients in the group of o-fLC negative samples, whereas no such correlation is observed when there is intrathecal fLC synthesis. (TIFF) [file pone.0166556.s003.tiff]

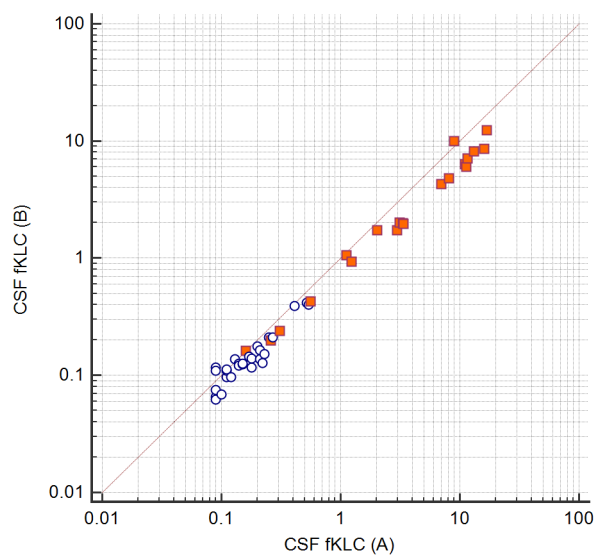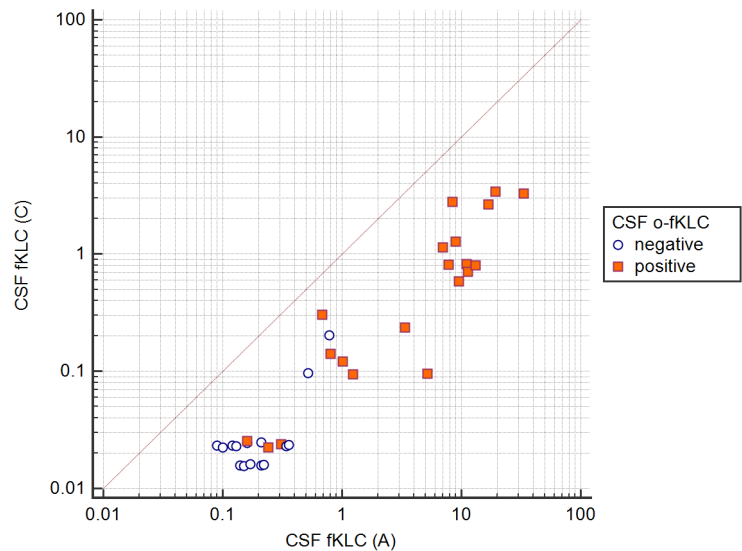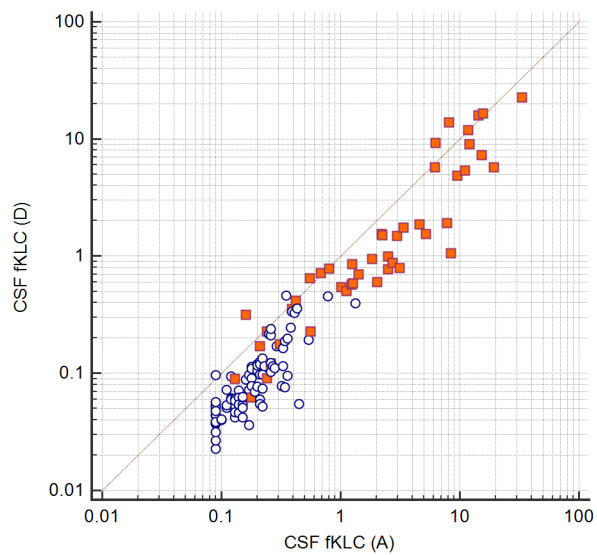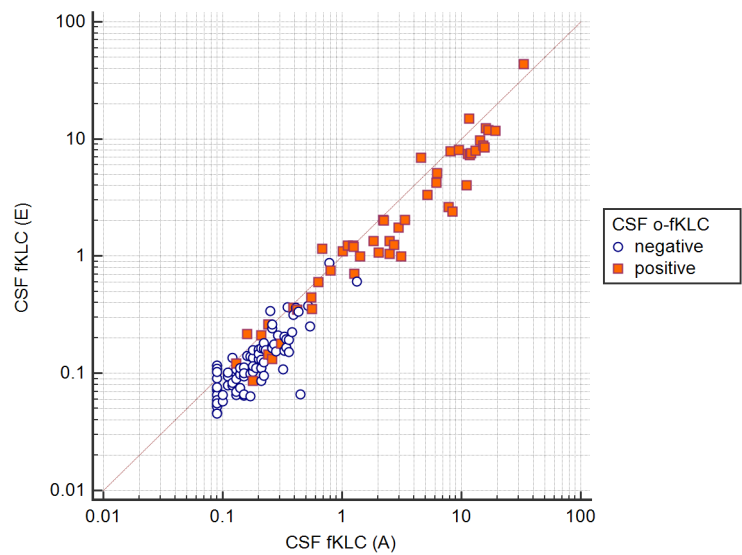

Supplement: S2 File — Method comparison (scatter diagrams). A. CSF fKLC (mg/L) B. Serum fKLC (mg/L) C. CSF fLLC (mg/L) D. Serum fLLC (mg/L) E. Q fKLC (∙ 103) F. Q fLLC (∙ 103) CSF, cerebrospinal fluid; fLC, free light chains; fKLC, free kappa light chains; fLLC, free lambda light chains; Q, CSF/Serum quotient. (A), Freelite™ assay on the SPAPLUS analyser; (B) N Latex FLC™ assay on BN ProSpec analyser; (C) commercially available ELISA (BioVendor); (D), in-house ELISA using monoclonal standards (Bethyl Laboratories); (E), in-house ELISA using Freelite™ standards (ZIP) [file pone.0166556.s005.zip › S2 Fig A.pdf]

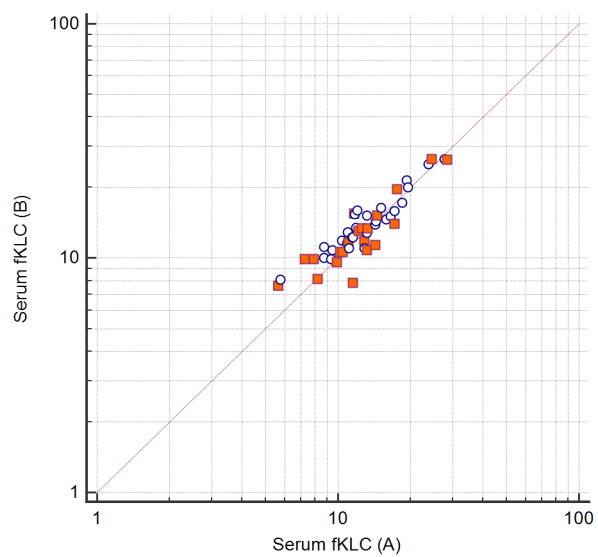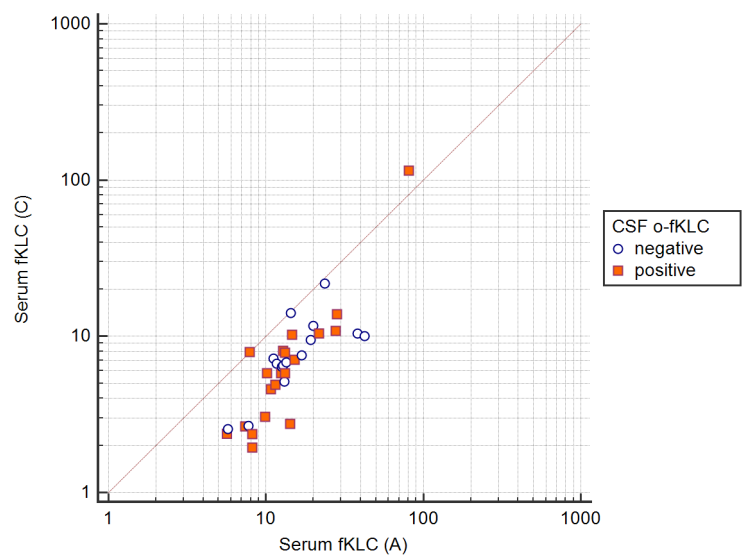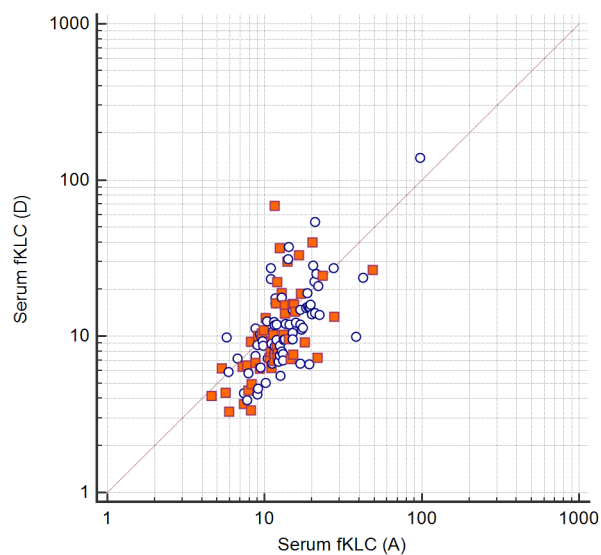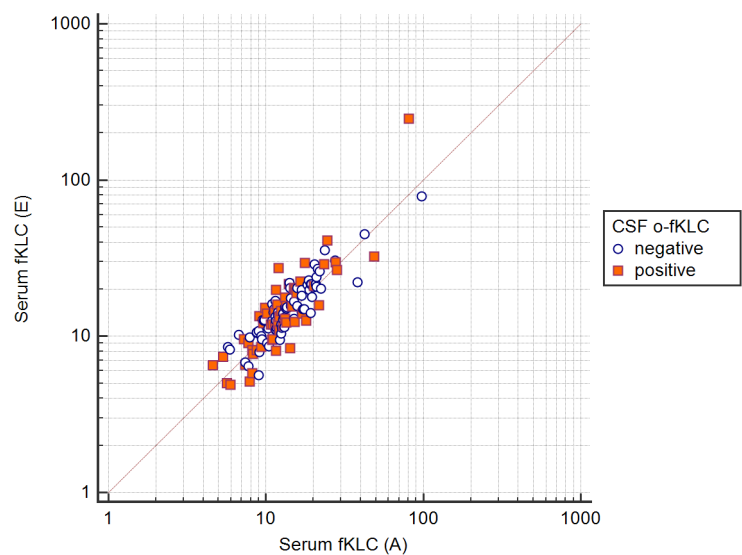

Supplement: S2 File — Method comparison (scatter diagrams). A. CSF fKLC (mg/L) B. Serum fKLC (mg/L) C. CSF fLLC (mg/L) D. Serum fLLC (mg/L) E. Q fKLC (∙ 103) F. Q fLLC (∙ 103) CSF, cerebrospinal fluid; fLC, free light chains; fKLC, free kappa light chains; fLLC, free lambda light chains; Q, CSF/Serum quotient. (A), Freelite™ assay on the SPAPLUS analyser; (B) N Latex FLC™ assay on BN ProSpec analyser; (C) commercially available ELISA (BioVendor); (D), in-house ELISA using monoclonal standards (Bethyl Laboratories); (E), in-house ELISA using Freelite™ standards (ZIP) [file pone.0166556.s005.zip › S2 Fig B.pdf]

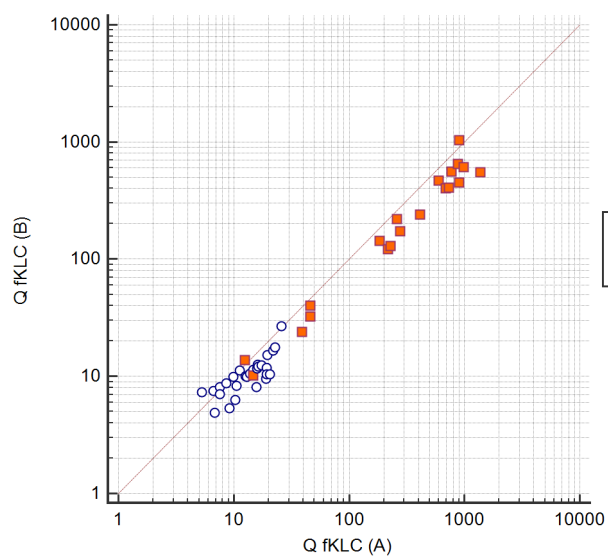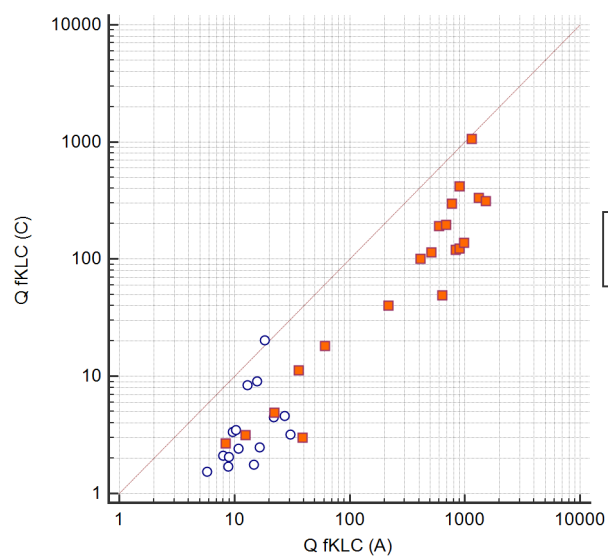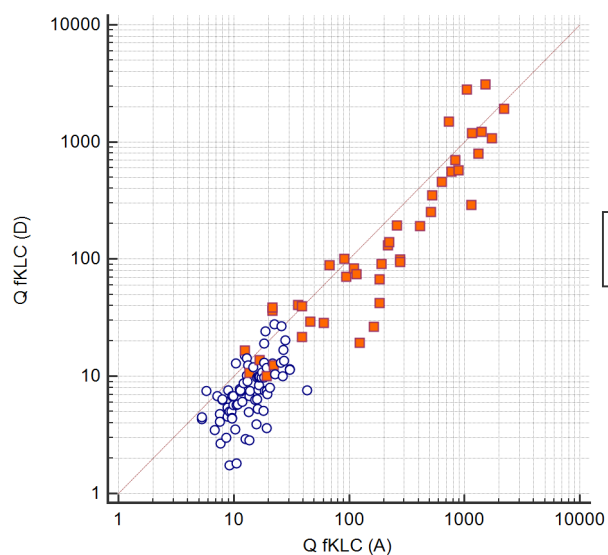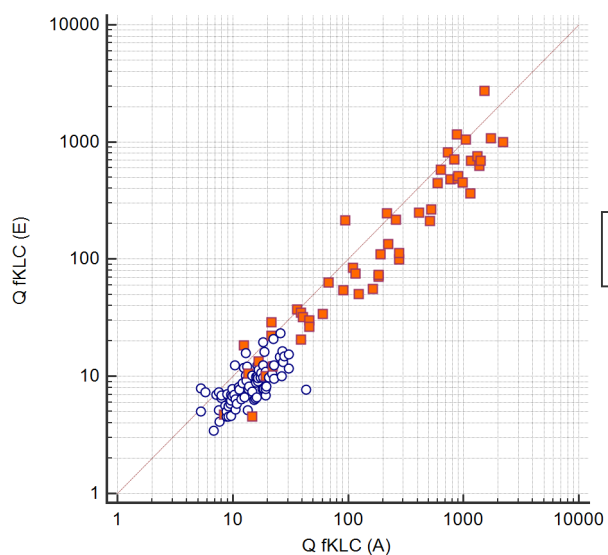

Supplement: S2 File — Method comparison (scatter diagrams). A. CSF fKLC (mg/L) B. Serum fKLC (mg/L) C. CSF fLLC (mg/L) D. Serum fLLC (mg/L) E. Q fKLC (∙ 103) F. Q fLLC (∙ 103) CSF, cerebrospinal fluid; fLC, free light chains; fKLC, free kappa light chains; fLLC, free lambda light chains; Q, CSF/Serum quotient. (A), Freelite™ assay on the SPAPLUS analyser; (B) N Latex FLC™ assay on BN ProSpec analyser; (C) commercially available ELISA (BioVendor); (D), in-house ELISA using monoclonal standards (Bethyl Laboratories); (E), in-house ELISA using Freelite™ standards (ZIP) [file pone.0166556.s005.zip › S2 Fig C.pdf]

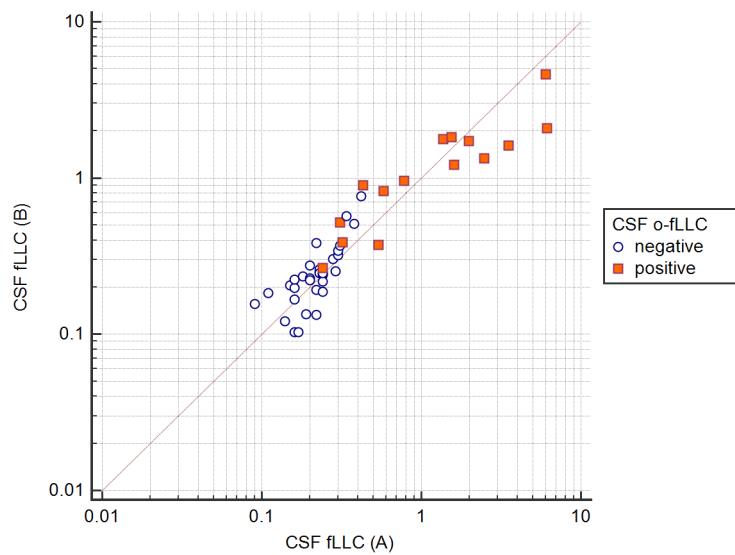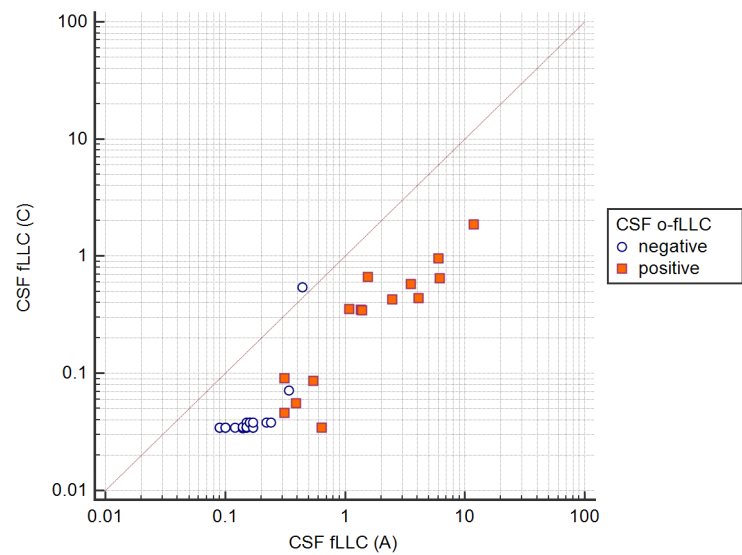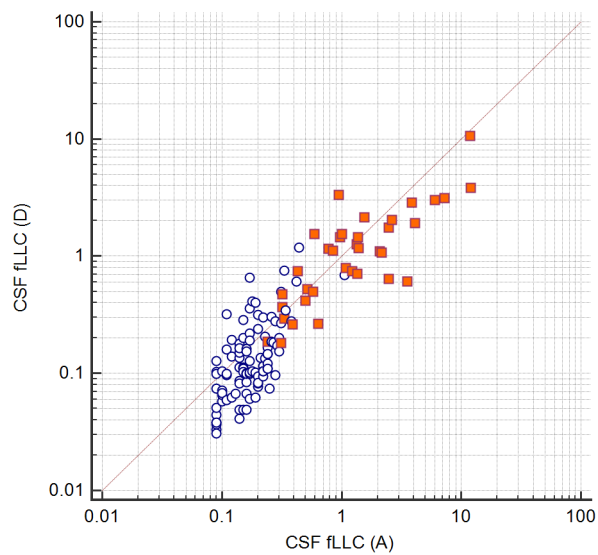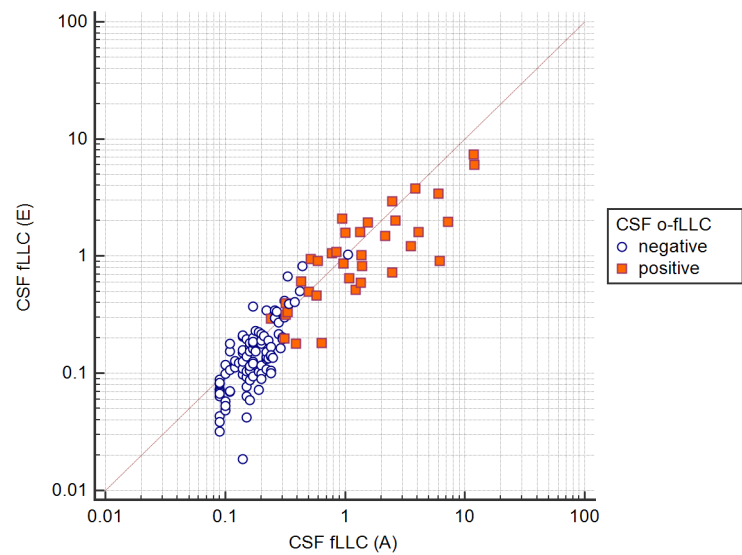

Supplement: S2 File — Method comparison (scatter diagrams). A. CSF fKLC (mg/L) B. Serum fKLC (mg/L) C. CSF fLLC (mg/L) D. Serum fLLC (mg/L) E. Q fKLC (∙ 103) F. Q fLLC (∙ 103) CSF, cerebrospinal fluid; fLC, free light chains; fKLC, free kappa light chains; fLLC, free lambda light chains; Q, CSF/Serum quotient. (A), Freelite™ assay on the SPAPLUS analyser; (B) N Latex FLC™ assay on BN ProSpec analyser; (C) commercially available ELISA (BioVendor); (D), in-house ELISA using monoclonal standards (Bethyl Laboratories); (E), in-house ELISA using Freelite™ standards (ZIP) [file pone.0166556.s005.zip › S2 Fig D.pdf]

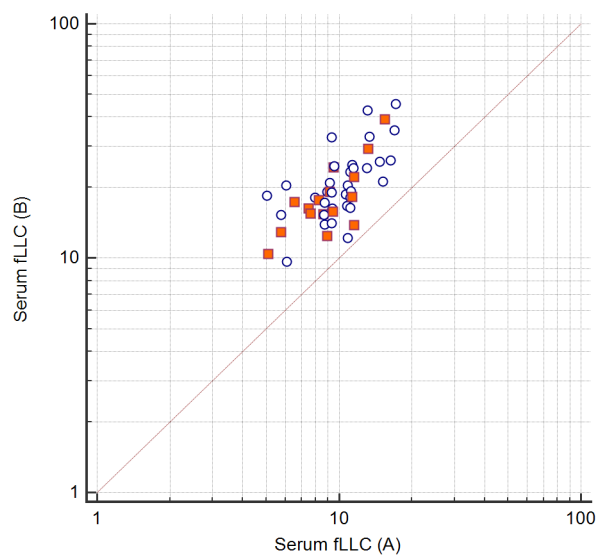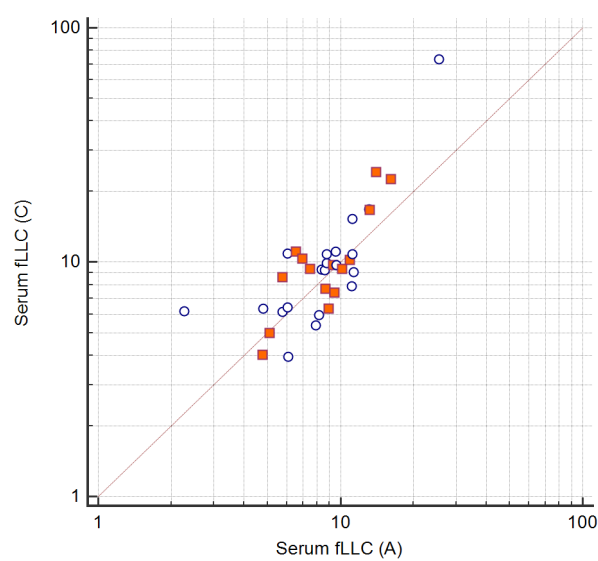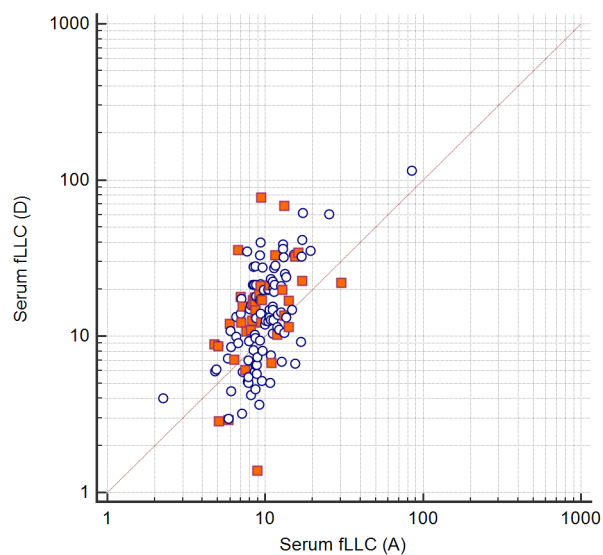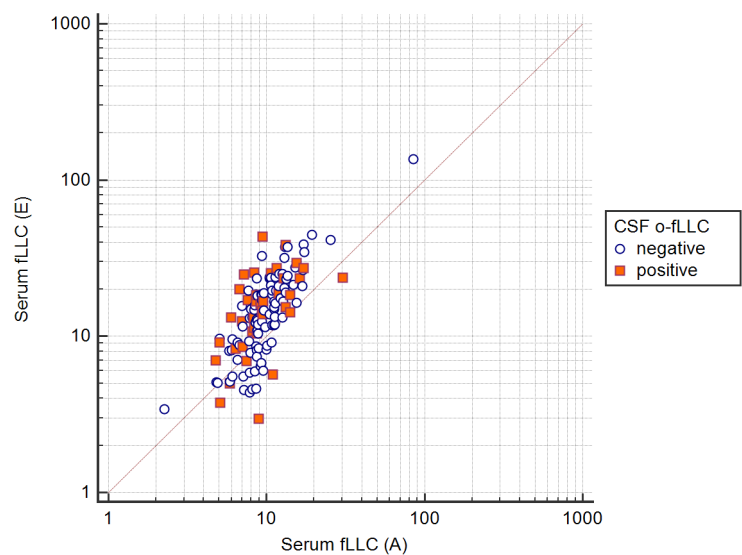

Supplement: S2 File — Method comparison (scatter diagrams). A. CSF fKLC (mg/L) B. Serum fKLC (mg/L) C. CSF fLLC (mg/L) D. Serum fLLC (mg/L) E. Q fKLC (∙ 103) F. Q fLLC (∙ 103) CSF, cerebrospinal fluid; fLC, free light chains; fKLC, free kappa light chains; fLLC, free lambda light chains; Q, CSF/Serum quotient. (A), Freelite™ assay on the SPAPLUS analyser; (B) N Latex FLC™ assay on BN ProSpec analyser; (C) commercially available ELISA (BioVendor); (D), in-house ELISA using monoclonal standards (Bethyl Laboratories); (E), in-house ELISA using Freelite™ standards (ZIP) [file pone.0166556.s005.zip › S2 Fig E.pdf]

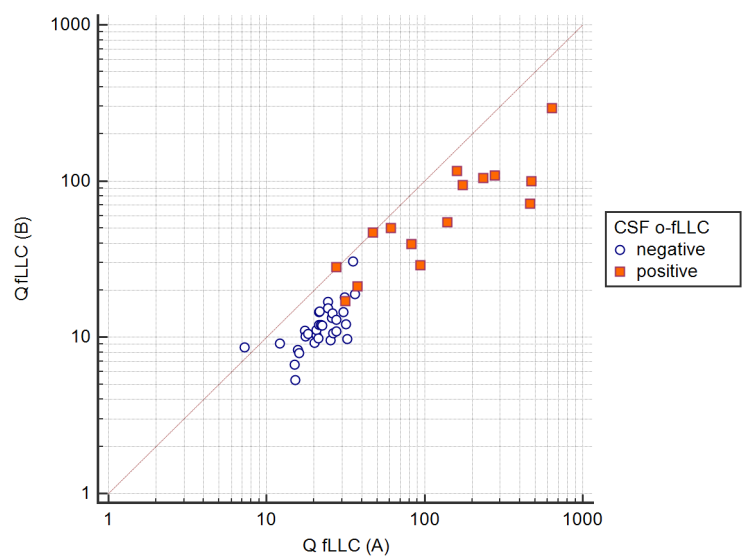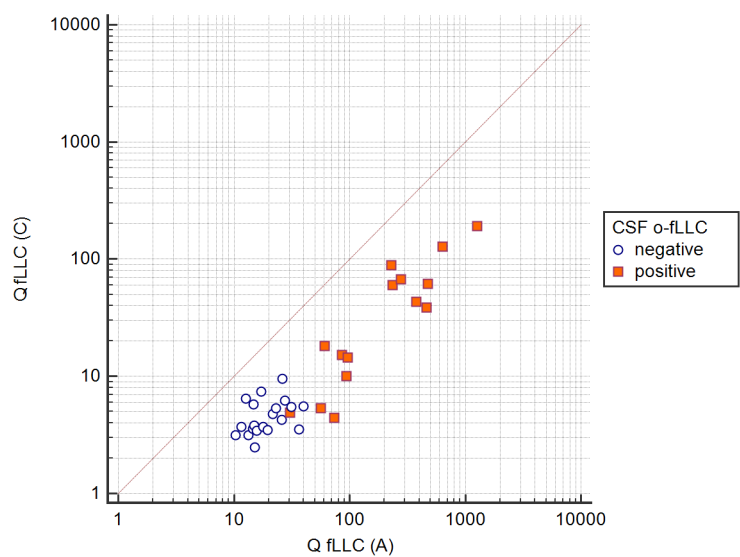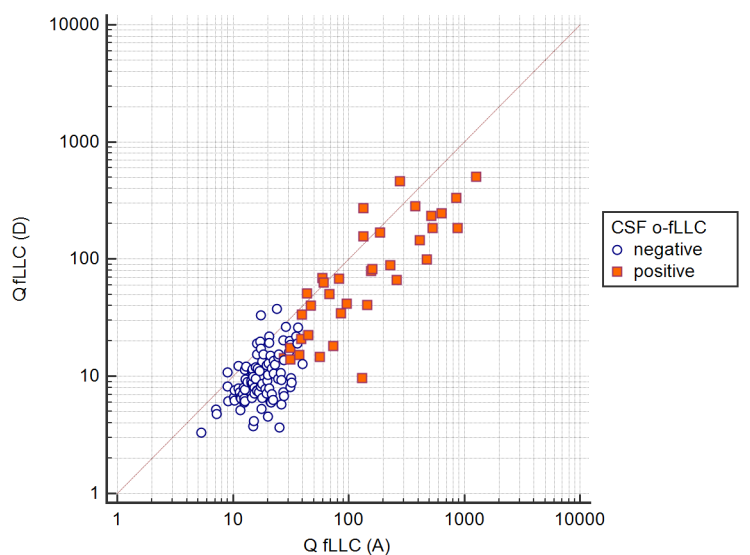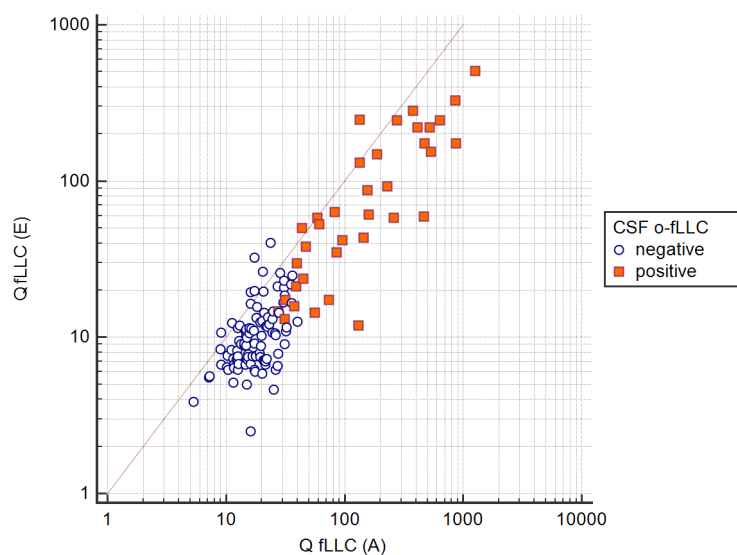

Supplement: S2 File — Method comparison (scatter diagrams). A. CSF fKLC (mg/L) B. Serum fKLC (mg/L) C. CSF fLLC (mg/L) D. Serum fLLC (mg/L) E. Q fKLC (∙ 103) F. Q fLLC (∙ 103) CSF, cerebrospinal fluid; fLC, free light chains; fKLC, free kappa light chains; fLLC, free lambda light chains; Q, CSF/Serum quotient. (A), Freelite™ assay on the SPAPLUS analyser; (B) N Latex FLC™ assay on BN ProSpec analyser; (C) commercially available ELISA (BioVendor); (D), in-house ELISA using monoclonal standards (Bethyl Laboratories); (E), in-house ELISA using Freelite™ standards (ZIP) [file pone.0166556.s005.zip › S2 Fig F.pdf]

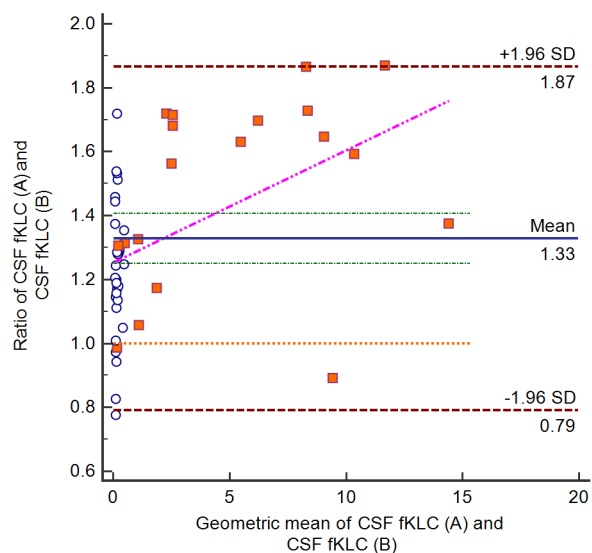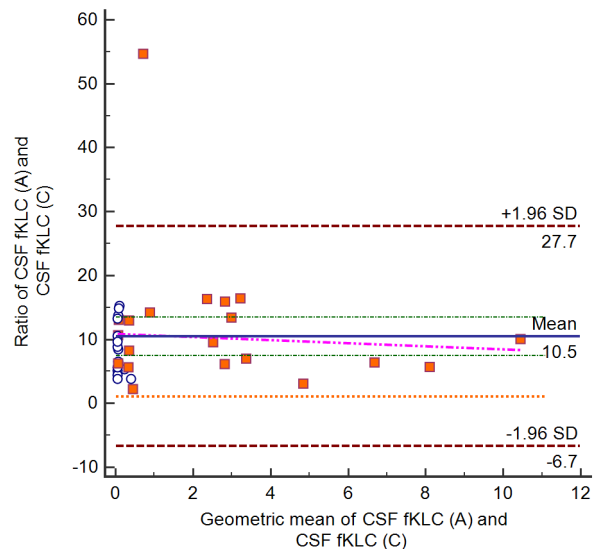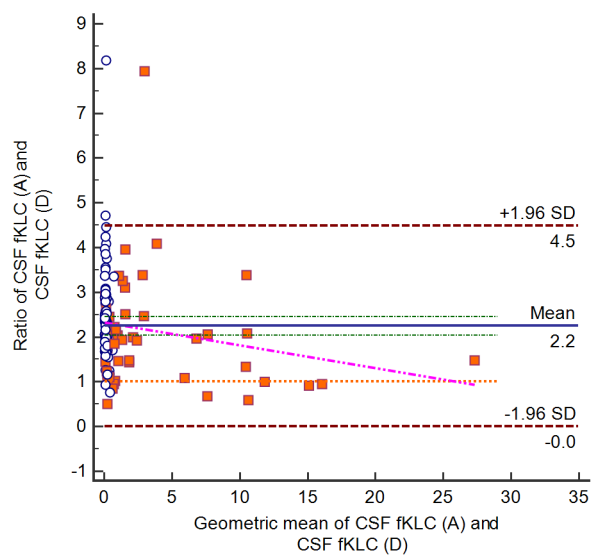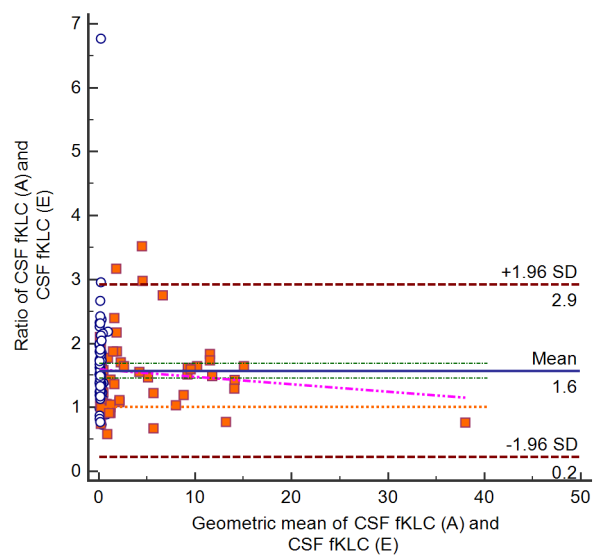

Supplement: S3 File — A. CSF fKLC (mg/L) B. Serum fKLC (mg/L) C. CSF fLLC (mg/L) D. Serum fLLC (mg/L) E. Q fKLC (∙ 103) F. Q fLLC (∙ 103) CSF, cerebrospinal fluid; fLC, free light chains; fKLC, free kappa light chains; fLLC, free lambda light chains; Q, CSF/Serum quotient. (A), Freelite™ assay on the SPAPLUS analyser; (B) N Latex FLC™ assay on BN ProSpec analyser; (C) commercially available ELISA (BioVendor); (D), in-house ELISA using monoclonal standards (Bethyl Laboratories); (E), in-house ELISA using Freelite™ standards. (ZIP) [file pone.0166556.s006.zip › S3 Fig A.pdf]

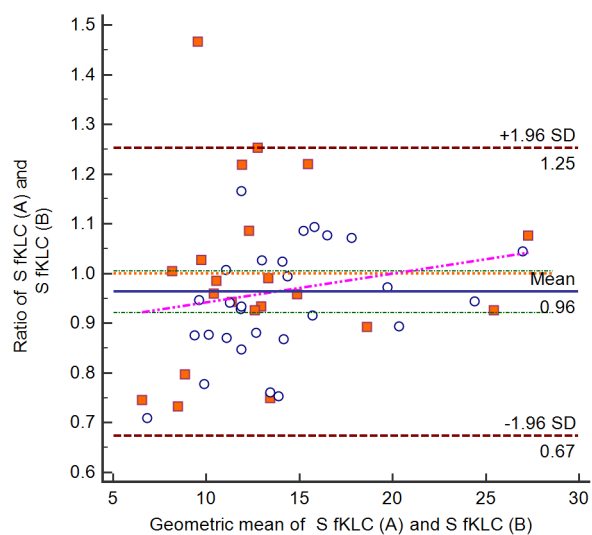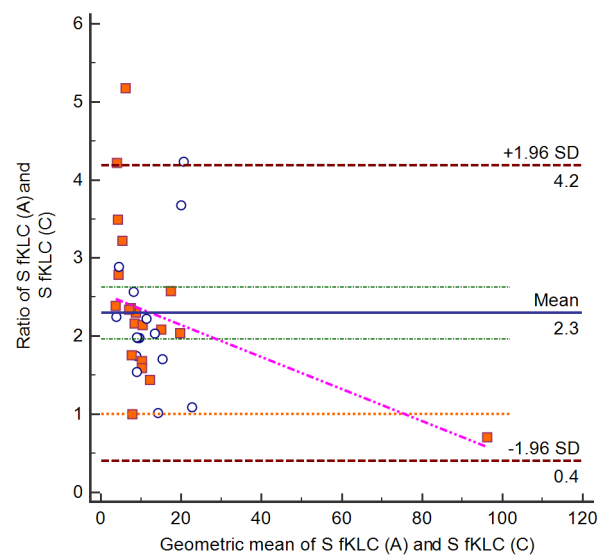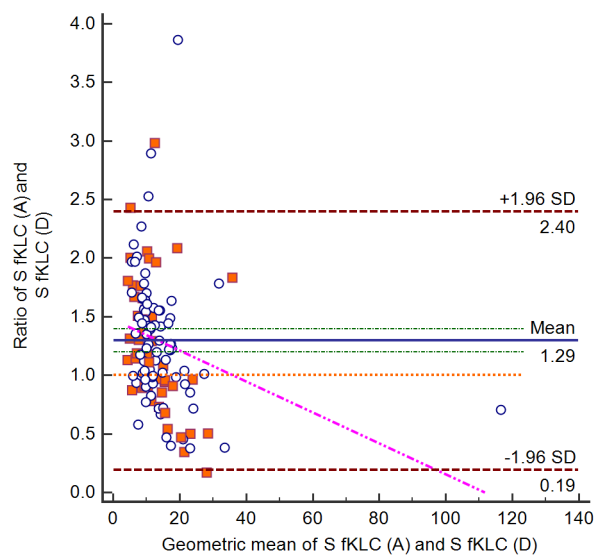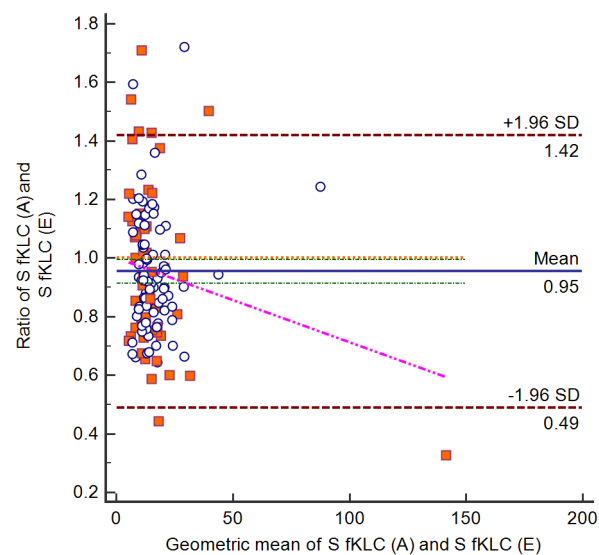

Supplement: S3 File — A. CSF fKLC (mg/L) B. Serum fKLC (mg/L) C. CSF fLLC (mg/L) D. Serum fLLC (mg/L) E. Q fKLC (∙ 103) F. Q fLLC (∙ 103) CSF, cerebrospinal fluid; fLC, free light chains; fKLC, free kappa light chains; fLLC, free lambda light chains; Q, CSF/Serum quotient. (A), Freelite™ assay on the SPAPLUS analyser; (B) N Latex FLC™ assay on BN ProSpec analyser; (C) commercially available ELISA (BioVendor); (D), in-house ELISA using monoclonal standards (Bethyl Laboratories); (E), in-house ELISA using Freelite™ standards. (ZIP) [file pone.0166556.s006.zip › S3 Fig B.pdf]

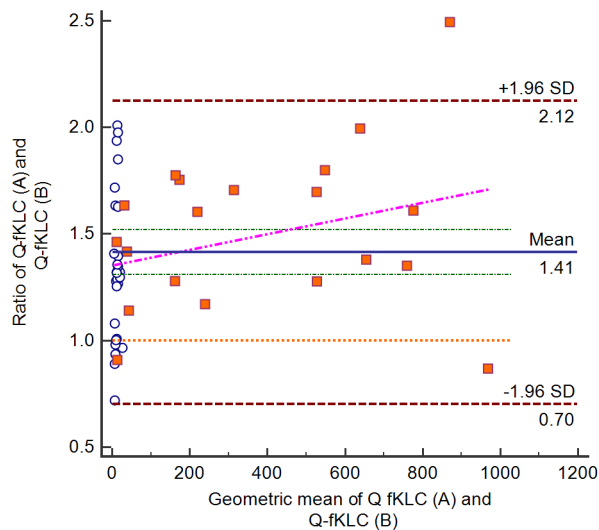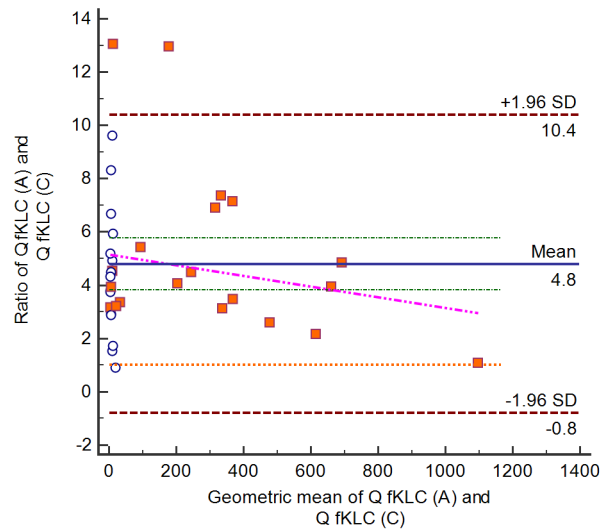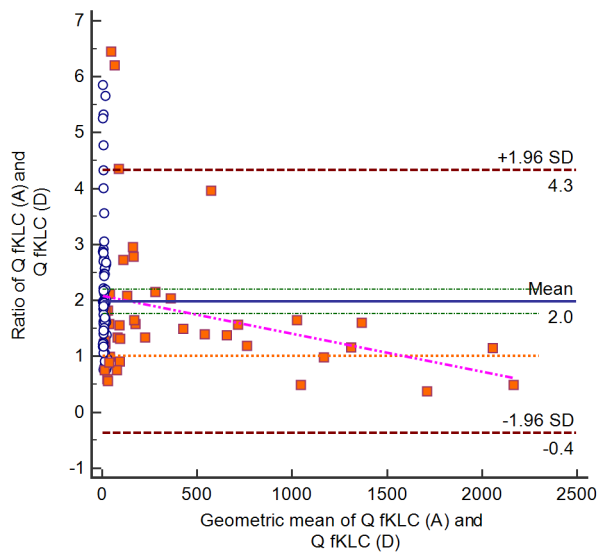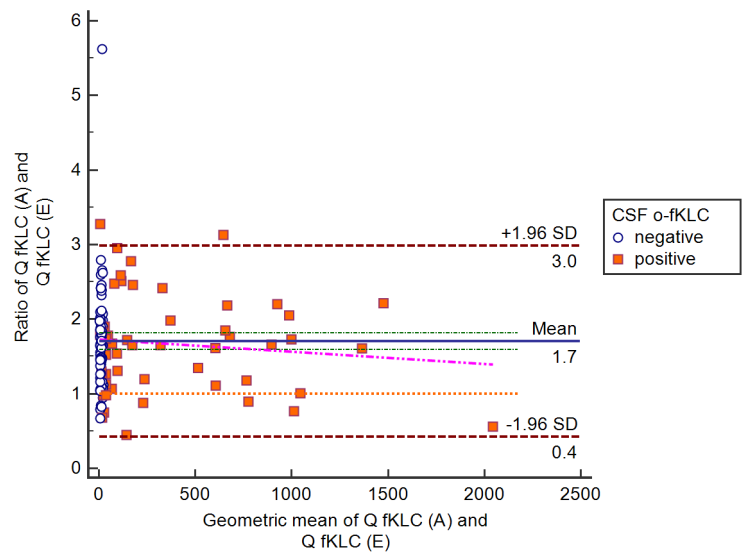

Supplement: S3 File — A. CSF fKLC (mg/L) B. Serum fKLC (mg/L) C. CSF fLLC (mg/L) D. Serum fLLC (mg/L) E. Q fKLC (∙ 103) F. Q fLLC (∙ 103) CSF, cerebrospinal fluid; fLC, free light chains; fKLC, free kappa light chains; fLLC, free lambda light chains; Q, CSF/Serum quotient. (A), Freelite™ assay on the SPAPLUS analyser; (B) N Latex FLC™ assay on BN ProSpec analyser; (C) commercially available ELISA (BioVendor); (D), in-house ELISA using monoclonal standards (Bethyl Laboratories); (E), in-house ELISA using Freelite™ standards. (ZIP) [file pone.0166556.s006.zip › S3 Fig C.pdf]

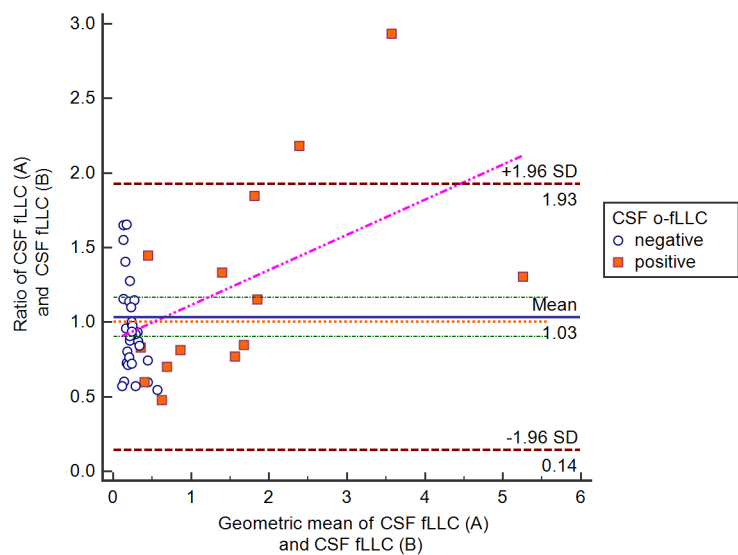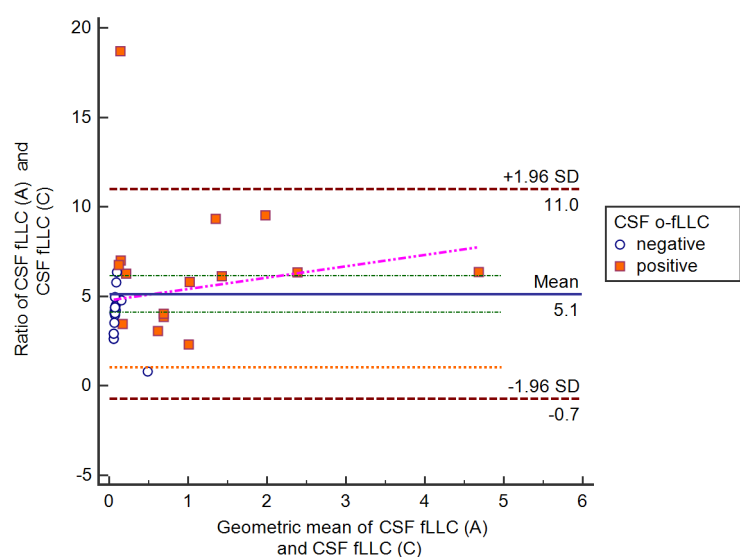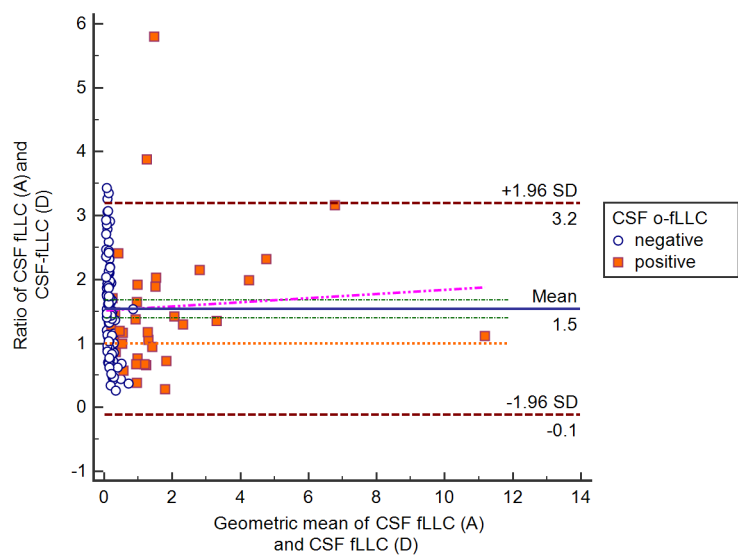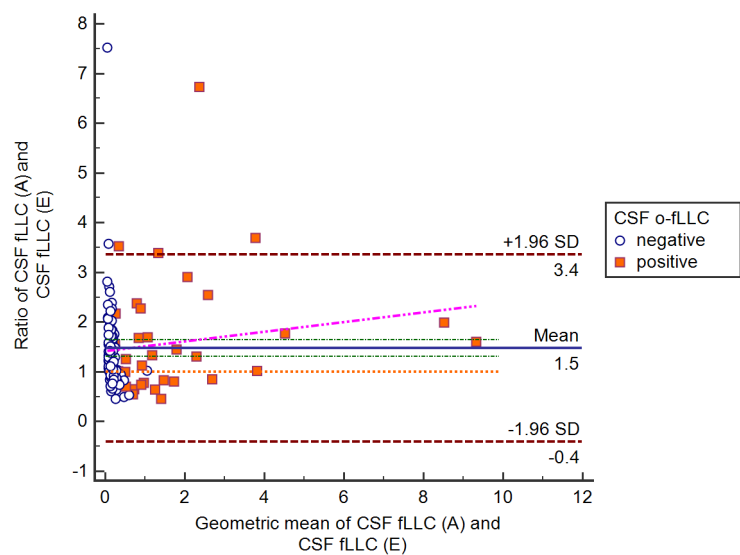

Supplement: S3 File — A. CSF fKLC (mg/L) B. Serum fKLC (mg/L) C. CSF fLLC (mg/L) D. Serum fLLC (mg/L) E. Q fKLC (∙ 103) F. Q fLLC (∙ 103) CSF, cerebrospinal fluid; fLC, free light chains; fKLC, free kappa light chains; fLLC, free lambda light chains; Q, CSF/Serum quotient. (A), Freelite™ assay on the SPAPLUS analyser; (B) N Latex FLC™ assay on BN ProSpec analyser; (C) commercially available ELISA (BioVendor); (D), in-house ELISA using monoclonal standards (Bethyl Laboratories); (E), in-house ELISA using Freelite™ standards. (ZIP) [file pone.0166556.s006.zip › S3 Fig D.pdf]

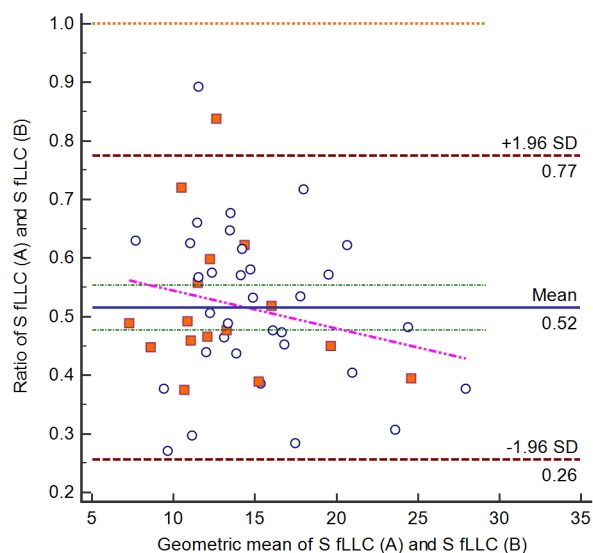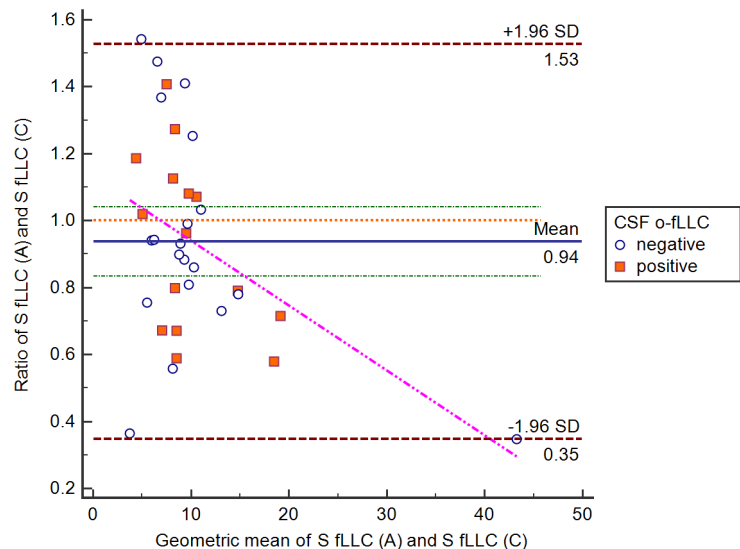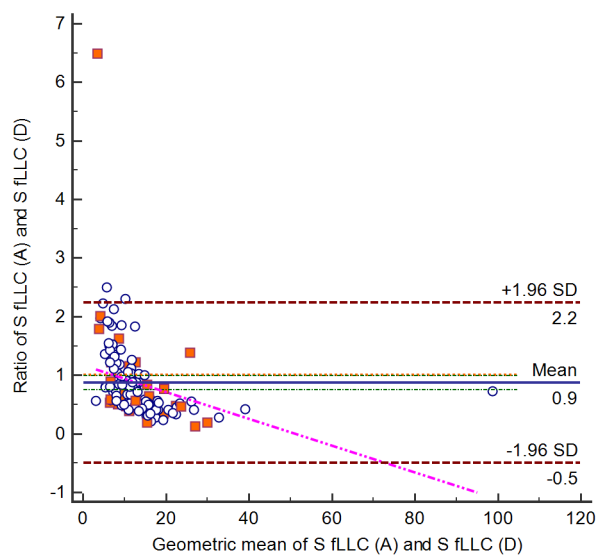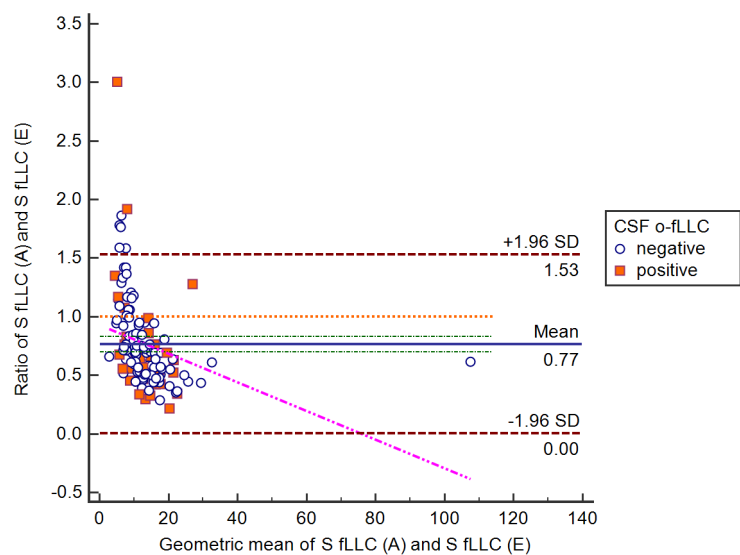

Supplement: S3 File — A. CSF fKLC (mg/L) B. Serum fKLC (mg/L) C. CSF fLLC (mg/L) D. Serum fLLC (mg/L) E. Q fKLC (∙ 103) F. Q fLLC (∙ 103) CSF, cerebrospinal fluid; fLC, free light chains; fKLC, free kappa light chains; fLLC, free lambda light chains; Q, CSF/Serum quotient. (A), Freelite™ assay on the SPAPLUS analyser; (B) N Latex FLC™ assay on BN ProSpec analyser; (C) commercially available ELISA (BioVendor); (D), in-house ELISA using monoclonal standards (Bethyl Laboratories); (E), in-house ELISA using Freelite™ standards. (ZIP) [file pone.0166556.s006.zip › S3 Fig E.pdf]

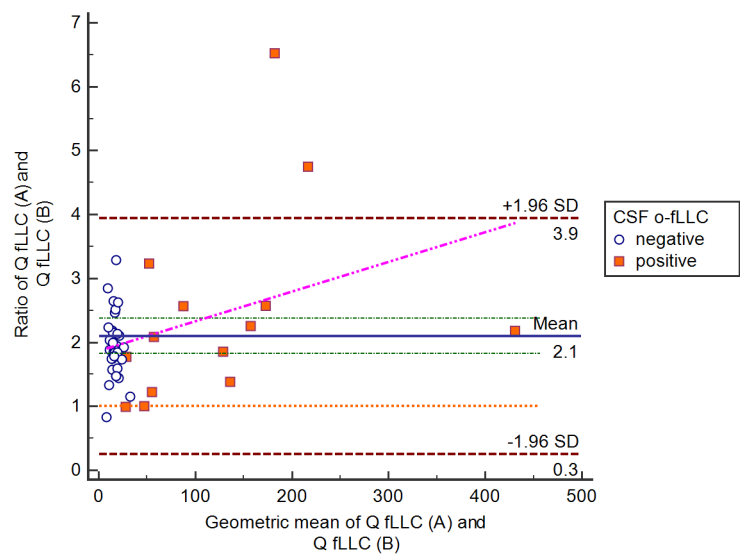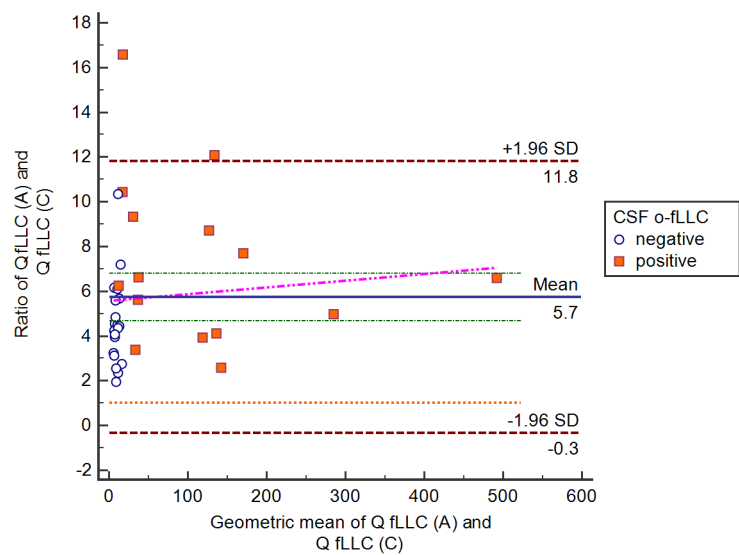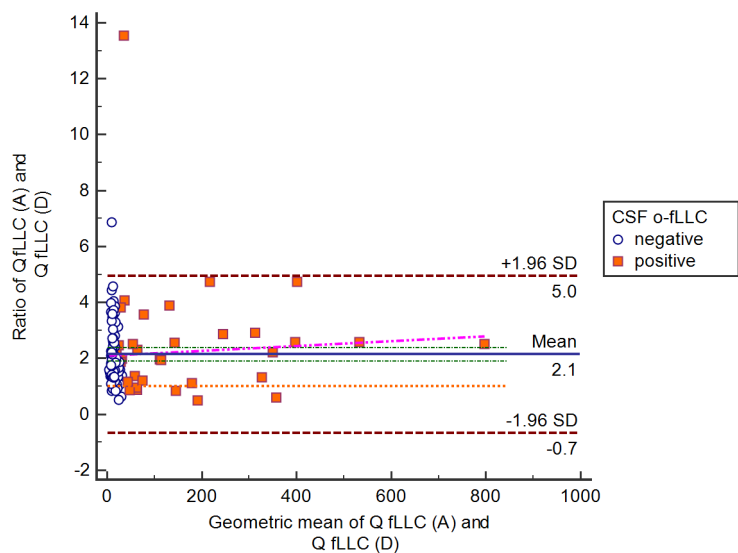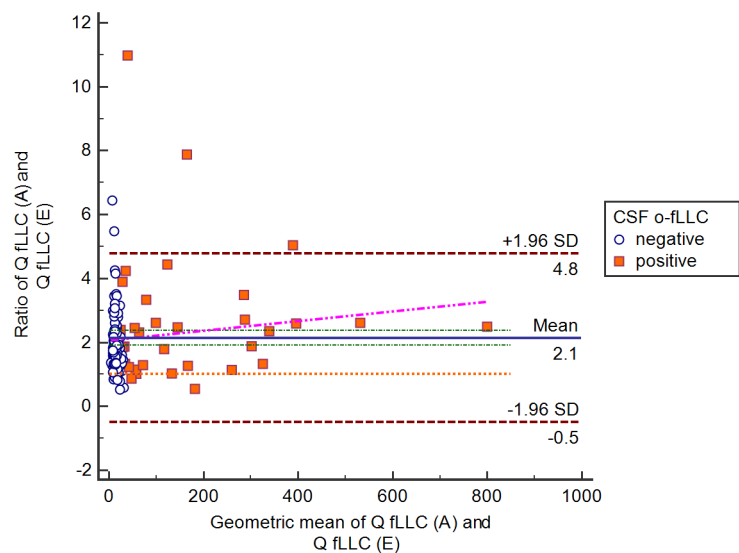

Supplement: S3 File — A. CSF fKLC (mg/L) B. Serum fKLC (mg/L) C. CSF fLLC (mg/L) D. Serum fLLC (mg/L) E. Q fKLC (∙ 103) F. Q fLLC (∙ 103) CSF, cerebrospinal fluid; fLC, free light chains; fKLC, free kappa light chains; fLLC, free lambda light chains; Q, CSF/Serum quotient. (A), Freelite™ assay on the SPAPLUS analyser; (B) N Latex FLC™ assay on BN ProSpec analyser; (C) commercially available ELISA (BioVendor); (D), in-house ELISA using monoclonal standards (Bethyl Laboratories); (E), in-house ELISA using Freelite™ standards. (ZIP) [file pone.0166556.s006.zip › S3 Fig F.pdf]
