# Supplementary material for: Assessment of Intrathecal Free Light Chain Synthesis: Comparison of Different Quantitative Methods with the Detection of Oligoclonal Free Light Chains by Isoelectric Focusing and Affinity-Mediated Immunoblotting
Source: PLoS One. 2016 Nov 15;11(11):e0166556. doi: 10.1371/journal.pone.0166556 (PMC5112955; doi:10.1371/journal.pone.0166556)
Supplement: S1 File — Table A. Comparison of CSF fLC concentrations measured by methods (A)—(E) by means of Passing and Bablok regression and Spearman´s correlation coefficient. (A), Freelite™ assay on the SPAPLUS analyser; (B) N Latex FLC™ assay on BN ProSpec analyser; (C) commercially available ELISA (BioVendor); (D), in-house ELISA using monoclonal standards (Bethyl Laboratories); (E), in-house ELISA using Freelite™ standards; fLC, free light chains; fKLC, free kappa light chains; fLLC, free lambda light chains; CI, confidence interval Table B. Comparison of serum fLC concentrations measured by methods (A)—(E) by means of Passing and Bablok regression and Spearman´s correlation coefficient. (A), Freelite™ assay on the SPAPLUS analyser; (B) N Latex FLC™ assay on BN ProSpec analyser; (C) commercially available ELISA (BioVendor); (D), in-house ELISA using monoclonal standards (Bethyl Laboratories); (E), in-house ELISA using Freelite™ standards; fLC, free light chains; fKLC, free kappa light chains; fLLC, free lambda light chains; CI, confidence interval Table C. Comparison of CSF/Serum fLC quotients by means of Passing and Bablok regression and Spearman´s correlation coefficient.(A), Freelite™ assay on the SPAPLUS analyser; (B) N Latex FLC™ assay on BN ProSpec analyser; (C) commercially available ELISA (BioVendor); (D), in-house ELISA using monoclonal standards (Bethyl Laboratories); (E), in-house ELISA using Freelite™ standards; fLC, free light chains; fKLC, free kappa light chains; fLLC, free lambda light chains; CI, confidence interval (ZIP) [file pone.0166556.s004.zip › Table A.rtf]

Table A. Comparison of cerebrospinal fluid free light chain concentrations measured by methods (A) - (E) by means of Passing and Bablok regression and Spearman´s correlation coefficient

a.	Ceresbrospinal fluid fKLC (mg/L)
	(A)	(B)	(A)	(C)	(A)	(D)	(A)	(E)	
n	49	35	127	134	
Lowest value	0.09	0.0623	0.09	0.0156	0.09	0.0227	0.09	0.0456	
Highest value	16.90	12.3000	33.20	3.4109	33.20	22.4800	33.20	43.4633	
Median	0.2500	0.1990	0.6800	0.0953	0.2500	0.1163	0.2600	0.1666	
Regression equation: y =	0.03316 + 0.6274 ∙ x	0.0173 + 0.08667 ∙ x	-0.01180 + 0.5433 ∙ x	-0.001692 + 0.6795 ∙ x	
Intercept (95% CI)	0.03316
(0.01489 – 0.04257)	0.0173
(-0.00390 – 0.01592)	-0.01180
(-0.03098 – -0.003656)	-0.001692
(-0.01572 – 0.008172)	
Slope (95% CI)	0.6274
(0.6025 – 0.7197)	0.08667
(0.06231 – 0.1300)	0.5433
(0.5014 – 0.6634)	0.6795
(0.6307 – 0.7517)	

Spearman´s rho (95% CI)	0.979
(0.962 – 0.988) P<0.0001	0.880
(0.774 – 0.938)
P<0.0001	0.924
(0.894 – 0.946)
P<0.0001	0.934
(0.909 – 0.953)
P<0.0001	


b.	Cerebrospinal fluid fLLC (mg/L)
	(A)	(B)	(A)	(C)	(A)	(D)	(A)	(E)	
n	48	35	135	135	
Lowest value	0.09	0.103	0.09	0.034	0.09	0.031	0.09	0.019	
Highest value	6.13	4.610	11.80	1.860	12.03	10.581	12.03	7.361	
Median	0.240	0.262	0.240	0.038	0.200	0.162	0.200	0.178	
Regression equation: y =	-0.02310 + 1.1962 ∙ x	0.01502 + 0.1449 ∙ x	-0.04327 + 0.9193 ∙ x	-0.03333 + 1.0159 ∙ x	
Intercept (95% CI)	-0.02310
(-0.07356 – 0.06120)	0.01502
(0.01219 – 0.02310)	-0.04327
(-0.07056 – -0.02237)	-0.03333
(-0.06531 – -0.008000)	
Slope (95% CI)	1.1962
(0.8423 – 1.3889)	0.1449
(0.1013 – 0.1601)	0.9193
(0.7967 – 1.1038)	1.0159
(0.8600 – 1.2034)	
Spearman´s rho
 (95% CI)	0.914
(0.851 – 0.951)
P<0.0001	0.864
(0.745 – 0.929)
P<0.0001	0.812
(0.746 – 0.863)
P<0.0001	0.863
(0.812 – 0.900)
P<0.0001	


(A), Freelite™ assay on the SPAPLUS analyser; (B) N Latex FLC™ assay on BN ProSpec analyser; (C) commercially available ELISA (BioVendor); (D), in-house ELISA using monoclonal standards (Bethyl Laboratories); (E), in-house ELISA using Freelite™ standards; fLC, free light chains; fKLC, free kappa light chains; fLLC, free lambda light chains; CI, confidence interval
