# Supplementary material for: Assessment of Intrathecal Free Light Chain Synthesis: Comparison of Different Quantitative Methods with the Detection of Oligoclonal Free Light Chains by Isoelectric Focusing and Affinity-Mediated Immunoblotting
Source: PLoS One. 2016 Nov 15;11(11):e0166556. doi: 10.1371/journal.pone.0166556 (PMC5112955; doi:10.1371/journal.pone.0166556)
Supplement: S1 File — Table A. Comparison of CSF fLC concentrations measured by methods (A)—(E) by means of Passing and Bablok regression and Spearman´s correlation coefficient. (A), Freelite™ assay on the SPAPLUS analyser; (B) N Latex FLC™ assay on BN ProSpec analyser; (C) commercially available ELISA (BioVendor); (D), in-house ELISA using monoclonal standards (Bethyl Laboratories); (E), in-house ELISA using Freelite™ standards; fLC, free light chains; fKLC, free kappa light chains; fLLC, free lambda light chains; CI, confidence interval Table B. Comparison of serum fLC concentrations measured by methods (A)—(E) by means of Passing and Bablok regression and Spearman´s correlation coefficient. (A), Freelite™ assay on the SPAPLUS analyser; (B) N Latex FLC™ assay on BN ProSpec analyser; (C) commercially available ELISA (BioVendor); (D), in-house ELISA using monoclonal standards (Bethyl Laboratories); (E), in-house ELISA using Freelite™ standards; fLC, free light chains; fKLC, free kappa light chains; fLLC, free lambda light chains; CI, confidence interval Table C. Comparison of CSF/Serum fLC quotients by means of Passing and Bablok regression and Spearman´s correlation coefficient.(A), Freelite™ assay on the SPAPLUS analyser; (B) N Latex FLC™ assay on BN ProSpec analyser; (C) commercially available ELISA (BioVendor); (D), in-house ELISA using monoclonal standards (Bethyl Laboratories); (E), in-house ELISA using Freelite™ standards; fLC, free light chains; fKLC, free kappa light chains; fLLC, free lambda light chains; CI, confidence interval (ZIP) [file pone.0166556.s004.zip › Table B.rtf]

Table B. Comparison of serum fLC concentrations measured by methods (A) - (E) by means of Passing and Bablok regression and Spearman´s correlation coefficient

a.	Serum fKLC (mg/L)
	(A)	(B)	(A)	(C)	(A)	(D)	(A)	(E)	
n	49	35	128	136	
Lowest value	5.67	7.60	5.670	1.936	4.63	3.302	4.6300	4.884	
Highest value	28.31	26.40	80.84	114.392	97.63	138.846	97.63	246.880	
Median	12.14	12.90	13.1400	6.8270	12.1100	9.8915	12.4600	13.7795	
Regression equation: y =	1.6975 + 0.2936 ∙ x	-1.7341 + 0.5943 ∙ x	-5.8200 + 1.2893 ∙ x	-1.9885 + 1.2415 ∙ x	
Intercept (95% CI)	1.6975
(0.02528 – 2.9964)	-1.7341
(-4.7082 – -0.1403)	-5.8200
(-9.0141 – -3.2059)	-1.9885
(-3.7575 – -0.3651)	
Slope (95% CI)	0.9236
(0.8119 – 1.0585)	0.5943
(0.4766 – 0.8610)	1.2893
(1.0671 – 1.5510)	1.2415
(1.1149 – 1.3981)	
Spearman´s rho 
 (95% CI)	0.865
(0.722 – 0.922)
P<0.0001	0.829
(0.686 – 0.911)
P<0.0001	0.647
(0.533 – 0.737)
P<0.0001	0.849
(0.794 – 0.890)
P<0.0001	


   b. Serum fLLC (mg/L)
	(A)	(B)	(A)	(C)	(A)	(D)	(A)	(E)	
n	49		35	136	136	
Lowest value	5.02	9.67	2.26	3.948	2.26	1.376	2.26	2.969	
Highest value	17.15	45.40	25.47	73.504	84.48	115.282	84.48	136.565	
Median	9.490	18.700	8.750	9.378	9.310	13.603	9.310	14.880	
Regression equation: 
y =	-7.0866 + 2.7515 ∙ x	-3.5037 + 1.5327 ∙ x	-24.2791 + 4.1292 ∙ x	-14.2741 + 3.0729 ∙ x	
Intercept (95% CI)	-7.0866
(-28.2554 – 0.2926)	-3.5037
(-8.7665 – 0.1190)	-24.2791
(-35.9720 – -16.6997)	-14.2741
(-20.2485 – -10.1233)	
Slope (95% CI)	2.7515
(2.0785 – 3.7404)	1.5327
(1.0607 – 2.0950)	4.1292
(3.3101 – 5.3814)	3.0729
(2.5952 – 3-6626)	
Spearman´s rho 
 (95% CI)	0.678
(0.490 – 0.806)
P<0.0001	0.691
(0.464 – 0,832)
P<0.0001	0.523
(0.389 – 0.636)
P<0.0001	0.696
(0.597 – 0.773)
P<0.0001	

(A), Freelite™ assay on the SPAPLUS analyser; (B) N Latex FLC™ assay on BN ProSpec analyser; (C) commercially available ELISA (BioVendor); (D), in-house ELISA using monoclonal standards (Bethyl Laboratories); (E), in-house ELISA using Freelite™ standards; fLC, free light chains; fKLC, free kappa light chains; fLLC, free lambda light chains; CI, confidence interval
