# Supplementary material for: Assessment of Intrathecal Free Light Chain Synthesis: Comparison of Different Quantitative Methods with the Detection of Oligoclonal Free Light Chains by Isoelectric Focusing and Affinity-Mediated Immunoblotting
Source: PLoS One. 2016 Nov 15;11(11):e0166556. doi: 10.1371/journal.pone.0166556 (PMC5112955; doi:10.1371/journal.pone.0166556)
Supplement: S1 File — Table A. Comparison of CSF fLC concentrations measured by methods (A)—(E) by means of Passing and Bablok regression and Spearman´s correlation coefficient. (A), Freelite™ assay on the SPAPLUS analyser; (B) N Latex FLC™ assay on BN ProSpec analyser; (C) commercially available ELISA (BioVendor); (D), in-house ELISA using monoclonal standards (Bethyl Laboratories); (E), in-house ELISA using Freelite™ standards; fLC, free light chains; fKLC, free kappa light chains; fLLC, free lambda light chains; CI, confidence interval Table B. Comparison of serum fLC concentrations measured by methods (A)—(E) by means of Passing and Bablok regression and Spearman´s correlation coefficient. (A), Freelite™ assay on the SPAPLUS analyser; (B) N Latex FLC™ assay on BN ProSpec analyser; (C) commercially available ELISA (BioVendor); (D), in-house ELISA using monoclonal standards (Bethyl Laboratories); (E), in-house ELISA using Freelite™ standards; fLC, free light chains; fKLC, free kappa light chains; fLLC, free lambda light chains; CI, confidence interval Table C. Comparison of CSF/Serum fLC quotients by means of Passing and Bablok regression and Spearman´s correlation coefficient.(A), Freelite™ assay on the SPAPLUS analyser; (B) N Latex FLC™ assay on BN ProSpec analyser; (C) commercially available ELISA (BioVendor); (D), in-house ELISA using monoclonal standards (Bethyl Laboratories); (E), in-house ELISA using Freelite™ standards; fLC, free light chains; fKLC, free kappa light chains; fLLC, free lambda light chains; CI, confidence interval (ZIP) [file pone.0166556.s004.zip › Table C.rtf]

Table C. Comparison of Cerebrospinal fluid/Serum fLC quotients by means of Passing and Bablok regression and Spearman´s correlation coefficient
a.	fKLC quotient (∙103)
	(A)	(B)	(A)	(C)	(A)	(D)	(A)	(E)	
n	48	35	121	133	
Lowest value	5.257	4.867	5.753	1.539	5.257	1.738	5.257	3.459	
Highest value	1373.494	1039.583	1524.334	1055.807	2197.393	3079.030	2197.393	2744.588	
Median	19.239	12.515	30.603	9.084	18.386	10.668	18.727	11.416	
Regression equation: 
y =	2.1470 + 0.6183 ∙ x	0.1518 + 0.2411 ∙ x	-1.4848 + 0.6782 ∙ x	0.1943 + 0.5878 ∙ x	
Intercept (95% CI)	2.1470
(0.4377 – 3.1564)	0.1518
(-0.8963 – 0.8395)	-1.4848
(-2.7191 – -0.5592)	0.1943
(-0.5115 – 1.0080)	
Slope (95% CI)		0.6183
	(0.5773 – 0.7246)	0.2411
(0.1864 – 0.2941)	0.6782
(0.6262 – 0.7526)	0.5878
(0.5482 – 0.6205)	
Spearman´s rho 
(95% CI)	0.947
(0.907 – 0.970)
P<0.0001	0.912
(0.832 – 0.955)
P<0.0001	0.877
(0.828 – 0.913)
P<0.0001	0.906
(0.870 – 0.932)
P<0.0001	


b.	fLLC quotient (∙103) 
	(A)	(B)	(A)	(C)	(A)	(D)	(A)	(E)	
n	47	35	134	134	
Lowest value	7.227	5.337	10.274	2.467	5.329	3.327	5.329	2.506	
Highest value	635.979	291.772	1263.383	191.712	1263.383	503.263	1263.383	506.517	
Median	26.273	14.174	30.572	5.528	21.328	11.614	21.328	11.617	
Regression equation:
y =	1.7152 + 0.4562 ∙ x	1.7015 + 0.1331 ∙ x	1.1327 + 0.4972 ∙ x	1.7301 + 0.4648 ∙ x	
Intercept (95% CI)	1.7152
(-0.9535 – 3.3531)	1.7015
(0.5903 – 2.5221)	1.1327
(-0.8201 – 2.5514)	1.7301
(-0.3208 – 2.9202)	
Slope (95% CI)	0.4562
(0.3895 – 0.5509)	0.1331
(0.1035 – 0.1813)	0.4972
(0.4184 – 0.6001)	0.4648
(0.3982 – 0.5602)	
Spearman´s rho
(95% CI)	0.894
(0.817 – 0.940)
P<0.0001	0.805
(0.645 – 0.898)
P<0.0001	0.757
(0.674 – 0.821)
P<0.0001	0.776
(0.699 – 0.836)
P<0.0001	
	
(A), Freelite™ assay on the SPAPLUS analyser; (B) N Latex FLC™ assay on BN ProSpec analyser; (C) commercially available ELISA (BioVendor); (D), in-house ELISA using monoclonal standards (Bethyl Laboratories); (E), in-house ELISA using Freelite™ standards; fLC, free light chains; fKLC, free kappa light chains; fLLC, free lambda light chains; CI, confidence interval
