# Supplementary material for: Assessment of Intrathecal Free Light Chain Synthesis: Comparison of Different Quantitative Methods with the Detection of Oligoclonal Free Light Chains by Isoelectric Focusing and Affinity-Mediated Immunoblotting
Source: PLoS One. 2016 Nov 15;11(11):e0166556. doi: 10.1371/journal.pone.0166556 (PMC5112955; doi:10.1371/journal.pone.0166556)
Supplement: S1 Table — a. according to the diagnosis group b. according to MS and IND status MS, multiple sclerosis; CIS, clinically isolated syndrome; OIND, other (than MS) inflammatory neurological diseases; NIND, non-inflammatory neurological diseases; IND, inflammatory neurological diseases. (RTF) [file pone.0166556.s007.rtf]

S1 Table. Results of o-IgG, o-fKLC and o-fLLC tests (≥2 CSF-restricted bands)
a.	according to the diagnosis group
Diagnosis group	o-IgG	o-fKLC	o-fLLC	
	negative	positive	negative	positive	negative	positive	
1 (MS)	4	13	2	15	8	9	
2 (CIS)	2	28	2	28	7	23	
3 (OIND)	14	6	9	11	11	9	
4 (NIND)	52	5	46	11	51	6	
5 (controls)	18	0	18	0	18	0	
9 (uncertain/ unknown)	19	5	15	9	18	6	


b.	according to MS and IND status 
	o-IgG	o-fKLC	o-fLLC	
	negative	positive	negative	positive	negative	positive	
MS	4	24	2	26	10	18	
Non-MS	84	11	73	22	80	15	
Chi-squared	54.798 (P<0.0001)	41.270 (P<0.0001)	23.497 (P<0.0001)	
IND	18	30	11	37	21	27	
NIND + controls	70	5	64	11	69	6	
Chi-squared	42.117 (P<0.0001)	45.332 (P<0.0001)	32.295 (P<0.0001)	

MS, multiple sclerosis; CIS, clinically isolated syndrome; OIND, other (than MS) inflammatory neurological diseases; NIND, non-inflammatory neurological diseases; IND, inflammatory neurological diseases
