# Supplementary material for: Assessment of Intrathecal Free Light Chain Synthesis: Comparison of Different Quantitative Methods with the Detection of Oligoclonal Free Light Chains by Isoelectric Focusing and Affinity-Mediated Immunoblotting
Source: PLoS One. 2016 Nov 15;11(11):e0166556. doi: 10.1371/journal.pone.0166556 (PMC5112955; doi:10.1371/journal.pone.0166556)
Supplement: S2 Table — a. Free kappa light chains b. Free lambda light chains MS, multiple sclerosis; non-MS, other diagnoses than multiple sclerosis or clinically isolated syndrome It should be noted that the number of samples examined by the N Latex FLC™ and BioVendor ELISA is too small to permit definitive conclusions regarding the performance of these tests in the context of MS diagnosis.* The second best cut-off value in case the calculated cut-off resulted in ≤50% sensitivity or specificity that was considered as unacceptable. (RTF) [file pone.0166556.s008.rtf]

S2 Table. Cut-offs in the context of multiple sclerosis diagnosis
a.	Free kappa light chains
	n
(MS/non-MS)	CSF fKLC
(mg/l)	fKLC quotient
(∙103)	fKLC index	
Freelite™ on SPAPLUS	26/92 (26/91 for fKLC quotient and index)	>0.78
(80.8; 87.0)	>46.1285
(84.6; 89.0)	>4.119
(92.3; 85.7)	
N Latex FLC™ on BN ProSpec	5/27	>1.72
(80.0; 100)	>122.1053
(80.0; 96.3)	>19.8421
(80.0; 96.3)	
ELISA (BioVendor)	5/20	>0.8145
(100; 95.0)	>114.7345
(100; 95.0)	>9.0217
(100; 100)	
ELISA (in-house, monoclonal standards)	15/84 (15/80 for fKLC quotient and index)	>0.3956
(86.7; 84.5)	>24.3275
(86.7; 83.8)	>3.0673
(86.7; 86.3)	
ELISA (in-house, Freelite™ standards)	17/86 (17/85 for fKLC quotient and index)	>0.6091
(82.4; 87.2)	>31.8283
(88.2; 90.6)	>3.0825
(88.2; 91.8)	


	
b.	Free lambda light chains
	n
(MS/non-MS)	CSF fLLC
(mg/l)	fLLC quotient
(∙103)	fLLC index	
Freelite™ on SPAPLUS	26/92
		>0.52
(57.7; 87.0)	>43.7342
(61.5; 88.0)	>7.5454
(65.4; 93.5)	
N Latex FLC™ on BN ProSpec	4/27	>0.899
(75.0; 96.3)	>21.1765
(100; 85.2)	>3.819
(100; 92.6)	
ELISA (BioVendor)	5/20	>0.0381
(80.0; 70.0)	>9.5745*
(60.0; 80.0)	>0.7895
(100; 60)	
ELISA (in-house, monoclonal standards)	18/86	>1.1029
(44.4; 95.4)	>22.0409*
(55.6; 84.9)	>3.411
(66.7; 91.9)	
ELISA (in-house, Freelite™ standards)	19/85	>0.6097
(57.9; 89.4)	>37.9521
(57.9; 90.6)	>3.223
(68.4; 90.6)	
MS, multiple sclerosis; non-MS, other diagnoses than multiple sclerosis or clinically isolated syndrome
It should be noted that the number of samples examined by the N Latex FLC™ and BioVendor ELISA is too small to permit definitive conclusions regarding the performance of these tests in the context of MS diagnosis.
* The second best cut-off value in case the calculated cut-off resulted in 50% sensitivity or specificity that was considered as unacceptable.
	
