# Supplementary material for: Assessment of Intrathecal Free Light Chain Synthesis: Comparison of Different Quantitative Methods with the Detection of Oligoclonal Free Light Chains by Isoelectric Focusing and Affinity-Mediated Immunoblotting
Source: PLoS One. 2016 Nov 15;11(11):e0166556. doi: 10.1371/journal.pone.0166556 (PMC5112955; doi:10.1371/journal.pone.0166556)
Supplement: S3 Table — a. Free kappa light chains b. Free lambda light chains IND, inflammatory neurological diseases; NIND, non-inflammatory neurological diseases and controls * The second best cut-off value in case the calculated cut-off resulted in ≤50% sensitivity or specificity that was considered as unacceptable. ** The second best cut-off value in case the calculated cut-off resulted in ≤50% specificity that was considered as unacceptable. (RTF) [file pone.0166556.s009.rtf]

S3 Table. Cut-offs in the context of inflammatory neurological disease diagnosis
a.	Free kappa light chains
	n
(IND/NIND)	CSF fKLC
(mg/l)	fKLC quotient
(∙103)	fKLC index	
Freelite™ on SPAPLUS	46/72	>0.35
(82.6; 84.7)	>26.9828
(71.7; 90.1))	>3.457
(80.4; 90.1)	
N Latex FLC™ on BN ProSpec	11/21	>0.399
(81.8; 100)	>26.7808
(72.7; 100)	>3.2021
(63.6; 100)	
ELISA (BioVendor)	14/11	>0.0953
(71.4; 100)	>3.3543
(92.9; 63.6)	>2.6088
(57.1; 100)	
ELISA (in-house, monoclonal standards)	33/66	>0.3546
(69.7; 92.4)	>12.8852
(80.0; 75.4)	>2.1971
(76.7; 86.2)	
ELISA (in-house, Freelite™ standards)	37/66	>0.3166
(75.7; 87.9)	>23.1889
(64.9; 93.9)	>3.0265
(67.6; 98.5)	


b.	Free lambda light chains
	n
(IND/NIND)	CSF fLLC
(mg/l)	fLLC quotient
(∙103)	fLLC index	
Freelite™ on SPAPLUS	46/72
	>0.31
(60.9; 87.5)	>36.3036
(56.5; 93.1)	>3.8432
(71.7; 81.9)	
N Latex FLC™ on BN ProSpec	10/21
	>0.368
(90.0; 85.7)	>18.8725
(80.0; 95.2)	>2.3798
(80.0; 81.0)	
ELISA (BioVendor)	14/11	>0.0381
(64.3; 90.9)	>9.5745**
(42.9; 90.9)	>0.7895
(78.6; 63.6)	
ELISA (in-house, monoclonal standards)	38/66	>0.287
(60.5; 81.8)	>13.4951
(73.7; 72.7)	>2.0476
(79.0; 77.3)	
ELISA (in-house, Freelite™ standards)	38/66	>0.4183*
(52.6; 92.4)	>13.4508
(71.1; 74.2)	>2.0177
(81.6; 77.3)	
IND, inflammatory neurological diseases; NIND, non-inflammatory neurological diseases and controls
* The second best cut-off value in case the calculated cut-off resulted in 50% sensitivity or specificity that was considered as unacceptable. 
** The second best cut-off value in case the calculated cut-off resulted in 50% specificity that was considered as unacceptable.
